# Phenyl palladium(II) iodide complexes isolated after Sonogashira coupling of iodo­benzenes with terminal alkynes

**DOI:** 10.1107/S2056989025004025

**Published:** 2025-05-13

**Authors:** Eric Bosch

**Affiliations:** a Chemistry and Biochemistry Department Missouri State University, 901 South national Avenue Springfield MO 65897 USA; Texas A & M University, USA

**Keywords:** crystal structure, oxidative addition, aryl palladium iodide

## Abstract

The structures of four palladium complexes serendipitously isolated after palladium-catalyzed Sonogashira coupling of aryl iodides with terminal alkynes are reported, namely [3,5-bis­(tri­fluoro­meth­yl)phen­yl]iodido­bis­(tri­phenyl­phosphane)palladium(II), [Pd(C_8_H_3_F_6_)I(C_18_H_15_P)_2_], **1**, (3,5-di­nitro­phen­yl)iodido­bis­(tri­phenyl­phosphane)palladium(II) ethyl acetate monosolvate, [Pd(C_6_H_3_N_2_O_4_)I(C_18_H_15_P)_2_]·C_4_H_8_O_2_, **2**, (2,3,4-di­fluoro­phen­yl)iodido­bis­(tri­phen­yl­phosphane)palladium(II), [Pd(C_6_H_2_F_3_)I(C_18_H_15_P)_2_], **3**, and (2,4,6-di­fluoro­phen­yl)iodido­bis­(tri­phenyl­phosphane)palladium(II) di­chloro­methane disolvate, [Pd(C_6_H_2_F_3_)I(C_18_H_15_P)_2_]·2CH_2_Cl_2_, **4**. These complexes were isolated as red/orange crystals that co-eluted with the organic products of each reaction during flash chromatography. These complexes are oxidative addition products ArPdI(PPh_3_)_2_ where Ar = 3,5-bis­(tri­fluoro­meth­yl)phenyl, 3,5-di­nitro­phenyl, 2,3,4-tri­fluoro­phenyl and 2,4,6-tri­fluoro-3,5-di­iodo­phenyl. The isolation of these complexes provides some insight into the possible fate of the palladium catalyst.

## Abstract 2

The structure of four *bis*(tri­phenyl­phosphine)phenyl­palladium(II) iodides isolated after completion of Sonogashira coupling reactions, in which the aryl iodide was used in slight excess, are reported.

## 1. Chemical context

**Figure 1:**
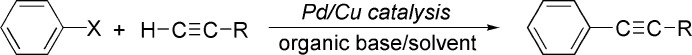
Reaction equation for Sonogashira coupling.

Palladium-catalyzed cross-coupling reactions are widely used in organic synthesis. The coupling reaction of aryl- and vinyl­halides with terminal alkynes to form alkynyl­benzenes is known as the Sonogashira Reaction (Sonogashira, 2002 ▸). A generic equation for the reaction with Cu co-catalysis is shown in Fig. 1 ▸ although it should be noted that the reaction can also be performed without copper co-catalysis. The generally accepted mechanism for the Sonogashira coupling involves oxidative addition of an aryl halide, Ar*X*, to a reactive palladium species to form ArPd*XL*_2_ where *L* is a neutral ligand such as triphenyl phosphine. With copper as cocatalyst, a copper acetyl­ide, CuCC*R*, is proposed to undergo transmetalation with ArPd*XL*_2_ to form a palladium complex with both the aryl (Ar) and acetyl­ide (CC*R*) groups bonded to the palladium center. Reductive elimination then produces the coupled product ArCC*R* and regenerates the active palladium catalyst Pd*L*_2_. In this report we present the structures of four palladium complexes incidentally isolated after Sonogashira coupling reactions. We noticed that, after column chromatography of the crude reaction product, the coupled products were often contaminated by trace amounts of red crystalline material. It is these ‘contaminants’ that are the subject of this study, providing insight into the fate of the palladium catalyst in these coupling reactions. It should be noted that, in these reactions, the aryl iodide was present in slight excess. Similar compounds have been isolated by stoichiometric reaction of an aryl halide with a palladium complex but this is, to our knowledge, the first X-ray characterization of an oxidative addition complex from a catalytic reaction.
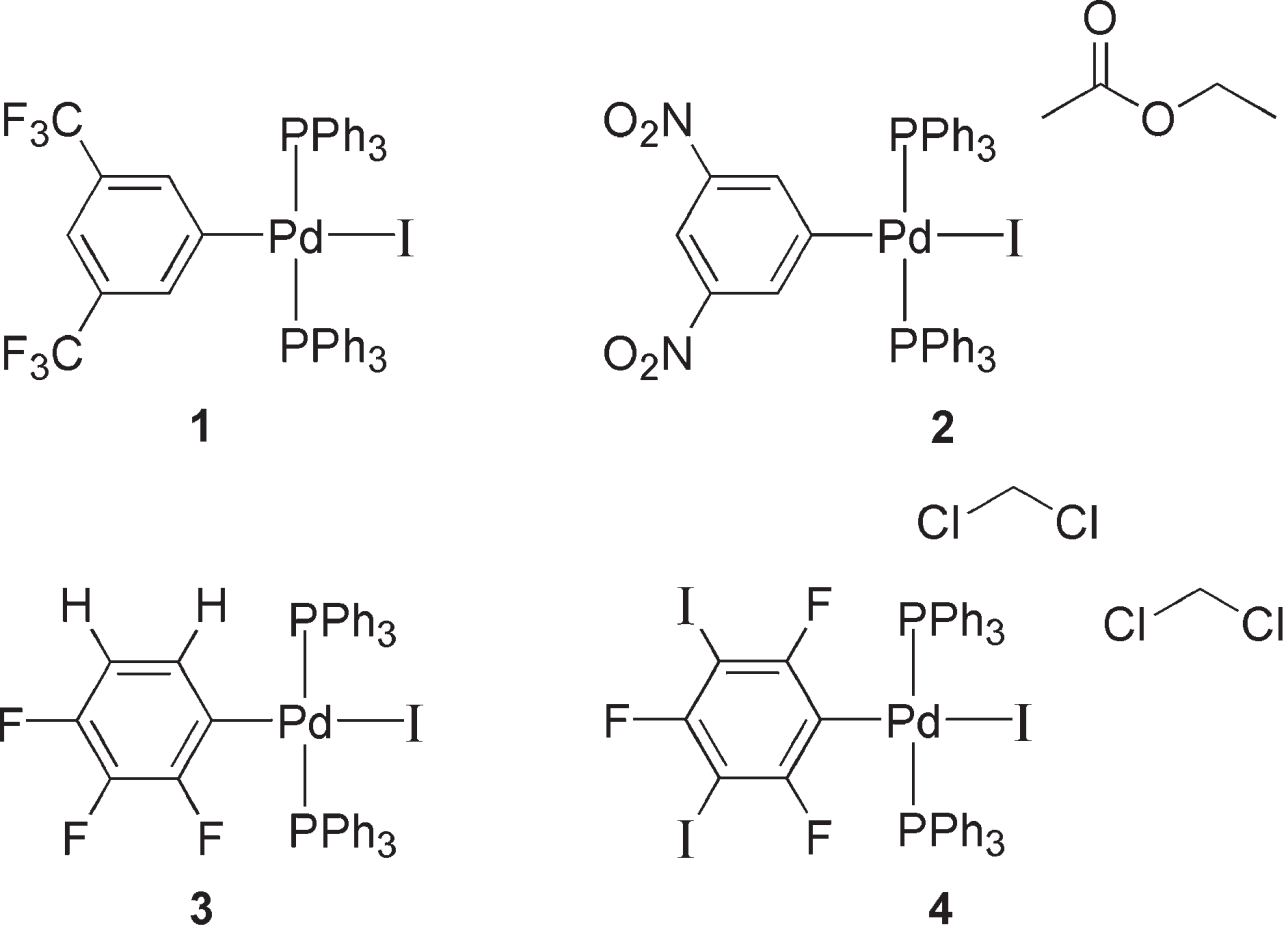


## 2. Structural commentary

**Figure 2:**
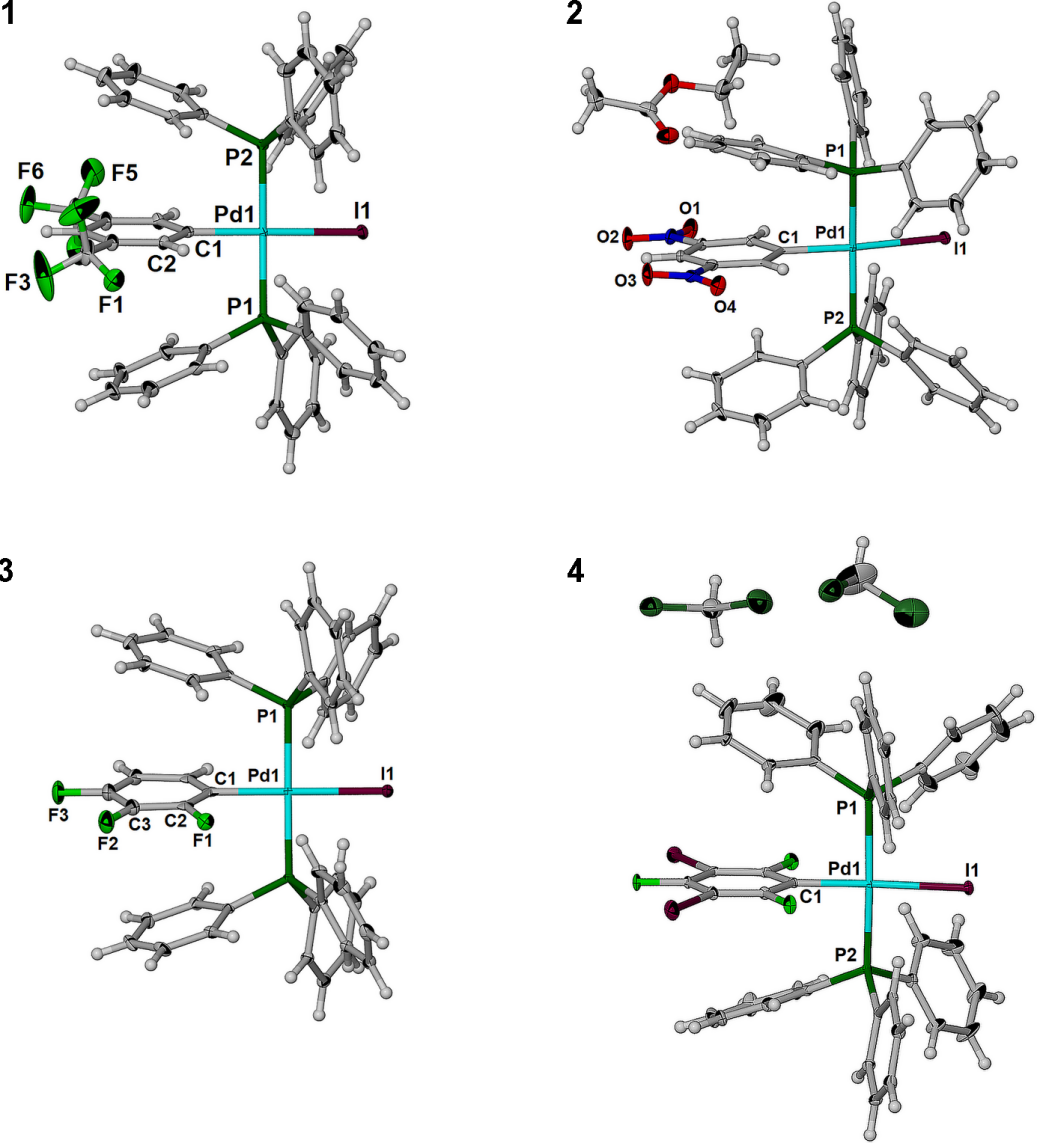
Asymmetric unit of each of the oxidative addition products **1**–**4** with displacement ellipsoids drawn at the 50% level. An ethyl acetate solvent mol­ecule is included in the ASU of **2** and two di­chloro­methane solvent mol­ecules, one disordered, are included in structure **4**. The major position of the disordered di­chloro­methane mol­ecule is shown.

The asymmetric unit of each of the structures is shown in Fig. 2 ▸. In each oxidative addition product **1**–**4**, the palladium has square-planar geometry with the aryl- and iodo- moieties in the *trans* configuration. The C—Pd—I and P—Pd—P angles are essentially linear in each structure, ranging from 171.82 (9) to 180° and from 171.36 (4) to 180°, respectively. Within these structures there is little deviation in the Pd1—I1, Pd1—C1 and Pd—P bond distances with ranges of 2.6626 (3) to 2.6887 (2) Å, 2.005 (4) to 2.088 (4) Å, and 2.3204 (6) to 2.3346 (10) Å, respectively. The C—Pd—P and I—Pd—P angles are clustered close to 90° in all four structures. All distances and angles are collated in Table 1 ▸.

## 3. Supra­molecular features

**Figure 3:**
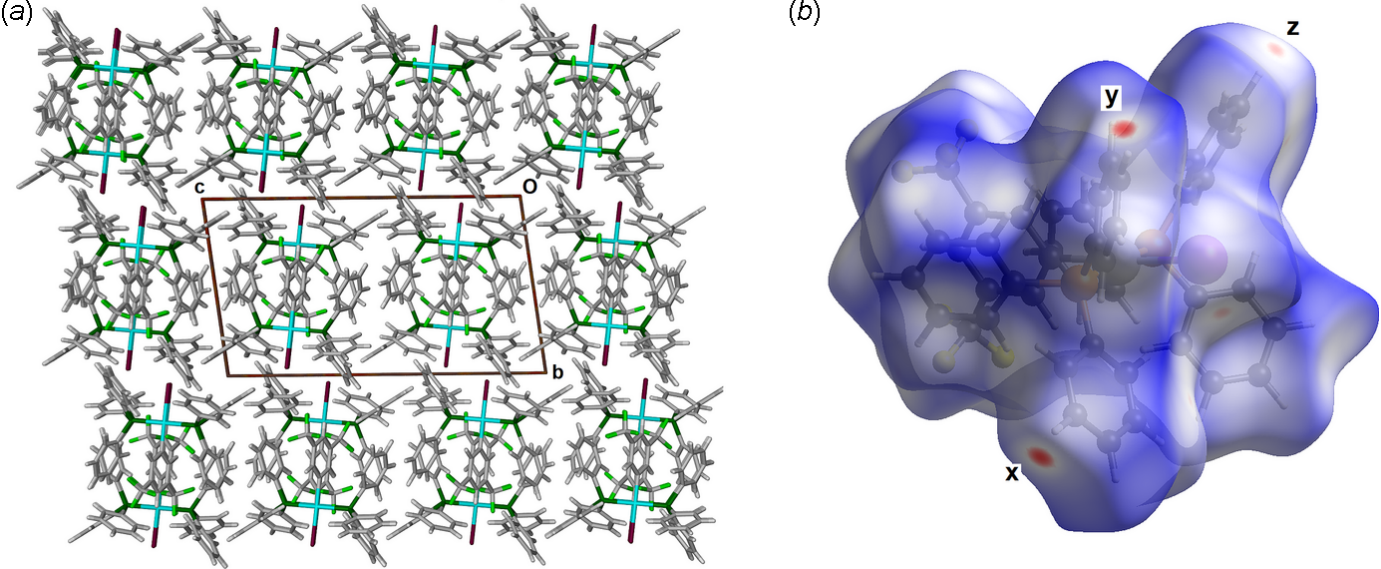
(*a*) View of the crystal packing in structure **1** viewed along the *a* axis. (*b*) Plot of the Hirshfeld surface of **1** within the crystal structure with close contacts labelled *x*, *y* and *z* (see text).

The unit-cell packing of **1** is shown in Fig. 3 ▸(*a*). The program *CrystalExplorer21* (Spackman *et al.*, 2021 ▸) was used to calculate the Hirshfeld surface of complex **1** within the crystal as shown in Fig. 3 ▸(*b*). The red areas labelled *x*, *y* and *z* in Fig. 3 ▸(*b*) correspond to contacts less than the sum of the van der Waals radii. Close contact *x* corresponds to the H43—H16^i^ inter­action [symmetry code: (i) *x*, −*y* + 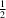, *z* + 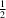] with separation 2.227 (4) Å. Contact *y* is the H35—F3^ii^ inter­action with separation 2.647 (4) Å [symmetry code: (ii) −*x* + 1, *y* + 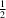, −*z* + 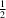] and contact *z* is a H18—H36^iii^ inter­action, separation 2.266 (4) Å [symmetry code: (iii) *x*, −*y* + 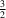, *z* − 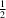]. These are likely not the result of attractive inter­molecular inter­actions between neighbouring mol­ecules, but rather the result of close packing. Fingerprint plots derived from the Hirshfeld surface provide a breakdown of the inter­molecular atom-to-atom contacts between atoms within the Hirshfeld surface and atoms outside the surface, including reciprocal inter­actions. Given the six phenyl rings on the periphery of the complex, it is unsurprising that the Hirshfeld fingerprint analysis reveals that the major atom-to-atom contact is H⋯H, corresponding to 48.3% of the surface area of a single complex **1**. With two peripheral tri­fluoro­methyl groups, F⋯H/H⋯F contacts are also abundant at 22.2% of the surface area. H⋯C/C⋯H contacts comprise 19.7% of the surface area. These results and those for complexes **2**, **3** and **4** are collated in Table 2 ▸. This analysis confirms that there are no strong attractive inter­molecular inter­actions between neighbouring complexes within each structure.

## 4. Database survey

A search of the Cambridge Crystallographic Database (Version 2024.3.0, build 426813; Groom *et al.*, 2016 ▸) using Conquest (Bruno *et al.*, 2002 ▸) for structures containing *bis*(tri­phenyl­phosphine)phenyl­palladium iodide, in which the substitution on the phenyl ring is not specified, yielded a total of 23 unique structures. These structures were synthesized by stoichiometric oxidative addition of an iodo­benzene to *tetra­kis*(tri­phenyl­phosphine)palladium, or a related palladium complex, for specific reactivity studies (Vicente *et al.*, 2004 ▸; Xu *et al.*, 2021 ▸). The bond distances and bond angles reported here for **1**–**4** (Table 1 ▸) are similar to those previously reported in these 23 structures. Thus the average C—Pd—I and P—Pd—P angles for these 23 structures are 172.70 and 172.84°, respectively, with average C—Pd and Pd—I distances 2.02 and 2.69 Å, respectively.

## 5. Synthesis and crystallization

The compounds **1**–**4** were isolated after Sonogashira coupling reactions between an aryl iodide with an alkyne in tri­ethyl­amine and bis­(tri­phenyl­phosphine)palladium(II) dichloride as catalyst and copper iodide as cocatalyst (Sonogashira, 2002 ▸). The aryl iodides 1-iodo-3,5-bis­(tri­fluoro­meth­yl)benzene, 1-iodo-3,5-di­nitro­benzene, 1,2,3-tri­fluoro-4-iodo­benzene and 1,3,5-tri­fluoro-2,4,6-tri­iodo­benzene were obtained from commercial sources and used as received. The alkynes used in these reactions were commercially available: 2-ethynyl­pyridine for **1** and **3**, and 5-ethynyl­pyrimidine, which remained from previous projects (Momose & Bosch, 2010 ▸), for **2** and **4**. In each of these reactions a slight excess of the aryl iodide was used. The product was detected as red/orange crystalline impurity in the bulk product isolated after rapid flash chromatography of the crude product after evaporation of the solvent with progressively more polar mixtures of hexane and ethyl acetate. Manual separation afforded small amounts of the complexes in crystalline form suitable for single-crystal X-ray crystallography.

## 6. Refinement

Crystal data, data collection and structure refinement details for complexes **1** – **4** are summarized in Table 3 ▸. All H atoms were observed in the difference maps during refinement and added to C as riding atoms in geometrically idealized positions with C—H = 0.95 Å (aromatic) and *U*_iso_(H) = 1.2*U*_eq_(C). In structure **1** there is evidence that tri­fluoro­methyl group (F1–F3) is disordered; however, this could not be satisfactorily resolved as potential alternate positions were observed in the difference map for only two of the three fluorine atoms. Accordingly, an ISOR command was used (F2 and F3). Crystals of **2** formed as a solvate with a single ethyl acetate mol­ecule in the asymmetric unit. During refinement of the structure of **2**, an *ortho* C atom, C2, on the di­nitro­benzene tended to NPD unless an EADP command (C1, C2) was included. In the structure of **3**, the tri­fluoroaryl group is disordered over two positions of equal occupancy that are related by a 180° rotation along the C1–C4 axis. Accordingly, EADP and EXYZ were used for C2, C2*A* and C3, C3*A*. Furthermore, the residual electron density of 2.05 that is 0.66 Å from C1 and 2.75 Å from Pd1 suggests that there is disorder between the position of the aryl moiety and the iodine atom. This residual electron density then affected refinement of C1 and required that the aryl moiety be refined with the aid of EADP command for atoms C1 and C4. Two di­chloro­methane solvent mol­ecules are included in the asymmetric unit of **4**. One of these is disordered over two major positions and these were refined with the help of a free variable that converged to 0.63087 after using a DFIX command of 1.79 (0.02) for the C—Cl distances.

## Supplementary Material

Crystal structure: contains datablock(s) 1, 2, 3, 4, global. DOI: 10.1107/S2056989025004025/jy2058sup1.cif

Structure factors: contains datablock(s) 1. DOI: 10.1107/S2056989025004025/jy20581sup2.hkl

Supporting information file. DOI: 10.1107/S2056989025004025/jy20581sup6.cdx

Structure factors: contains datablock(s) 2. DOI: 10.1107/S2056989025004025/jy20582sup3.hkl

Supporting information file. DOI: 10.1107/S2056989025004025/jy20582sup7.cdx

Structure factors: contains datablock(s) 3. DOI: 10.1107/S2056989025004025/jy20583sup4.hkl

Supporting information file. DOI: 10.1107/S2056989025004025/jy20583sup8.cdx

Structure factors: contains datablock(s) 4. DOI: 10.1107/S2056989025004025/jy20584sup5.hkl

Supporting information file. DOI: 10.1107/S2056989025004025/jy20584sup9.cdx

CCDC references: 2448820, 2448819, 2448818, 2448817

Additional supporting information:  crystallographic information; 3D view; checkCIF report

## Figures and Tables

**Table 1:** Distances and angles (Å, °) in the structures of **1**–**4**

Property	**1**	**2**	**3**	**4**
I1—Pd1	2.6887 (2)	2.6715 (4)	2.6787 (3)	2.6626 (3)
Pd1—P1	2.3206 (6)	2.3240 (10)	2.3239 (5)	2.3221 (9)
Pd1—C1	2.019 (2)	2.005 (4)	2.091 (4)	2.014 (3)
Pd1—P2	2.3240 (6)	2.3347 (10)	2.3239 (5)	2.3203 (9)
P2—Pd1—P1	173.08 (2)	171.36 (4)	179.47 (3)	171.80 (3)
C1—Pd1—I1	176.51 (7)	172.63 (10)	180.0	171.82 (9)
C1—Pd1—P1	89.90 (7)	89.35 (10)	90.263 (13)	89.09 (9)
P2—Pd1—I1	90.764 (15)	91.17 (3)	89.737 (13)	91.72 (2)
P2—Pd1—C1	89.57 (7)	90.00 (10)	90.263 (13)	88.63 (9)
P1—Pd1—I1	90.186 (15)	90.58 (3)	89.737 (13)	91.66 (2)

**Table 2:** Hirshfeld surface analysis of each complex **1**–**4** with an element-by-element delineation of the percentage contribution, including reciprocal contacts

Complex	H⋯H	H⋯C/C⋯H	H⋯F/F⋯H	C⋯C	C⋯F/F⋯C	H⋯I/I⋯H	I⋯F/F⋯I
**1**	48.3	22.2	19.7	1.5	1.5	4.7	1.0
**2**	49.4	21.1	21.5^*a*^	0	–	4.0	–
**3**	48.7	20.9	18.1	2.8	2.4	6.2	0.5
**4**	36.8	16.4	11.8	0.2	0.9	19.3	0.1

Note: (*a*) H⋯O/O⋯H contacts corresponding to the nitro groups.

**Table 3:** Experimental details

	**1**	**2**	**3**	**4**
Crystal data				
Chemical formula	[Pd(C_8_H_3_F_6_)I(C_18_H_15_P)_2_]	[Pd(C_6_H_3_N_2_O4)I(C_18_H_15_P)_2_]·C_4_H_8_O_2_	[Pd(C_6_H_2_F_3_)I(C_18_H_15_P)_2_]	[Pd(C_6_H_2_F_3_)I(C_18_H_15_P)_2_]·2CH_2_Cl_2_
*M*_r_	971.01	1013.12	888.99	1310.64
Crystal system, space group	Monoclinic, *P*2_1_/*c*	Monoclinic, *P*2_1_/*c*	Monoclinic, *I*2/*a*	Triclinic, *P* 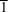
Temperature (K)	100	100	100	100
*a*, *b*, *c* (Å)	12.4498 (5), 14.5714 (6), 22.1464 (10)	11.7692 (7), 34.870 (2), 10.4381 (6)	11.6327 (5), 12.8059 (5), 23.4327 (9)	11.5608 (5), 11.6437 (5), 18.1311 (8)
α, β, γ (°)	90, 97.726 (1), 90	90, 99.920 (1), 90	90, 93.218 (2), 90	76.426 (1), 77.722 (1), 81.344 (1)
*V* (Å^3^)	3981.1 (3)	4219.7 (4)	3485.2 (2)	2305.16 (17)
*Z*	4	4	4	2
Radiation type	Mo *K*α	Mo *K*α	Mo *K*α	Mo *K*α
μ (mm^−1^)	1.38	1.30	1.56	2.76
Crystal size (mm)	0.2 × 0.2 × 0.2	0.4 × 0.25 × 0.1	0.28 × 0.27 × 0.23	0.23 × 0.11 × 0.10

Data collection				
Diffractometer	Bruker APEXI CCD	Bruker APEXI CCD	Bruker APEXI CCD	Bruker APEXI CCD
Absorption correction	Multi-scan (*SADABS*; Krause *et al.*, 2015 ▸)	Multi-scan (*SADABS*; Krause *et al.*, 2015 ▸)	Multi-scan (*SADABS*; Krause *et al.*, 2015 ▸)	Multi-scan (*SADABS*; Krause *et al.*, 2015 ▸)
*T*_min_, *T*_max_	0.703, 0.746	0.663, 0.746	0.705, 0.746	0.674, 0.746
No. of measured, independent and observed [*I* ≥ 2u(*I*)] reflections	50557, 8829, 8155	53507, 9320, 7734	22427, 3875, 3735	28953, 10132, 8450
*R*_int_	0.027	0.073	0.018	0.030
(sin θ/λ)_max_ (Å^−1^)	0.643	0.642	0.643	0.642

Refinement				
*R*[*F*^2^ > 2σ(*F*^2^)], *wR*(*F*^2^), *S*	0.028, 0.068, 1.03	0.045, 0.091, 1.06	0.021, 0.052, 1.05	0.030, 0.062, 1.06
No. of reflections	8829	9320	3875	10132
No. of parameters	487	519	229	542
No. of restraints	12	0	0	2
H-atom treatment	H-atom parameters constrained	H-atom parameters constrained	H-atom parameters constrained	H-atom parameters constrained
Δρ_max_, Δρ_min_ (e Å^−3^)	1.47, −1.17	1.50, −1.32	2.05, −1.12	1.06, −1.03

Computer programs: *OLEX2.solve* and *OLEX2.refine* (Bourhis *et al.*, 2015 ▸), *OLEX2* (Dolomanov *et al.*, 2009 ▸), *SHELXT* (Sheldrick, 2015 ▸) and *X-SEED* 4 (Barbour, 2020 ▸).

## supplementary crystallographic information

### (1) Crystal data

**Table:**

[Pd(C_8_H_3_F_6_)I(C_18_H_15_P)_2_]	*F*(000) = 1916.447
*M**_r_* = 971.01	*D*_x_ = 1.620 Mg m^−^^3^
Monoclinic, *P*2_1_/*c*	Mo *K*α radiation, λ = 0.71073 Å
*a* = 12.4498 (5) Å	Cell parameters from 9932 reflections
*b* = 14.5714 (6) Å	θ = 2.3–27.2°
*c* = 22.1464 (10) Å	µ = 1.38 mm^−^^1^
β = 97.726 (1)°	*T* = 100 K
*V* = 3981.1 (3) Å^3^	Cube, clear red
*Z* = 4	0.2 × 0.2 × 0.2 mm

### (1) Data collection

**Table:**

Bruker APEXI CCD diffractometer	8155 reflections with *I*≥ 2u(*I*)
φ and ω scans	*R*_int_ = 0.027
Absorption correction: multi-scan (SADABS; Krause *et al.*, 2015)	θ_max_ = 27.2°, θ_min_ = 1.7°
*T*_min_ = 0.703, *T*_max_ = 0.746	*h* = −16→15
50557 measured reflections	*k* = −18→18
8829 independent reflections	*l* = −28→28

### (1) Refinement

**Table:**

Refinement on *F*^2^	66 constraints
Least-squares matrix: full	Primary atom site location: iterative
*R*[*F*^2^ > 2σ(*F*^2^)] = 0.028	H-atom parameters constrained
*wR*(*F*^2^) = 0.068	*w* = 1/[σ^2^(*F*_o_^2^) + (0.0307*P*)^2^ + 8.078*P*] where *P* = (*F*_o_^2^ + 2*F*_c_^2^)/3
*S* = 1.02	(Δ/σ)_max_ = −0.001
8829 reflections	Δρ_max_ = 1.47 e Å^−^^3^
487 parameters	Δρ_min_ = −1.17 e Å^−^^3^
12 restraints	

### (1) Fractional atomic coordinates and isotropic or equivalent isotropic displacement parameters (Å^2^)

**Table:**

	*x*	*y*	*z*	*U*_iso_*/*U*_eq_	
I1	0.953448 (12)	0.427718 (10)	0.310946 (7)	0.01685 (5)	
Pd1	0.738937 (13)	0.403556 (11)	0.280333 (7)	0.01142 (5)	
P1	0.75605 (5)	0.43206 (4)	0.17898 (3)	0.01270 (11)	
F1	0.42586 (14)	0.19004 (12)	0.14520 (8)	0.0364 (4)	
C1	0.57665 (19)	0.39363 (16)	0.25832 (10)	0.0157 (4)	
P2	0.72222 (5)	0.35632 (4)	0.37887 (3)	0.01365 (11)	
C2	0.52665 (19)	0.31934 (16)	0.22612 (11)	0.0163 (4)	
H2	0.56971 (19)	0.27070 (16)	0.21386 (11)	0.0195 (5)*	
C3	0.4143 (2)	0.31576 (17)	0.21175 (12)	0.0215 (5)	
F4	0.34414 (17)	0.61366 (13)	0.24281 (11)	0.0534 (6)	
C4	0.3495 (2)	0.38559 (19)	0.22879 (12)	0.0228 (5)	
H4	0.2729 (2)	0.38266 (19)	0.21892 (12)	0.0274 (6)*	
F5	0.36128 (18)	0.56763 (15)	0.33453 (10)	0.0527 (6)	
C5	0.3979 (2)	0.45965 (18)	0.26040 (12)	0.0229 (5)	
F6	0.22636 (15)	0.52208 (15)	0.26987 (13)	0.0613 (7)	
C6	0.51119 (19)	0.46379 (17)	0.27507 (11)	0.0195 (5)	
H6	0.54341 (19)	0.51528 (17)	0.29675 (11)	0.0234 (6)*	
C7	0.3633 (2)	0.2336 (2)	0.17918 (16)	0.0354 (7)	
C8	0.3318 (2)	0.5391 (2)	0.27750 (16)	0.0360 (7)	
C9	0.85351 (18)	0.34876 (16)	0.15738 (10)	0.0148 (4)	
C10	0.8432 (2)	0.25782 (17)	0.17649 (11)	0.0207 (5)	
H10	0.7845 (2)	0.24137 (17)	0.19765 (11)	0.0249 (6)*	
C11	0.9176 (2)	0.19181 (17)	0.16484 (12)	0.0246 (5)	
H11	0.9097 (2)	0.13036 (17)	0.17788 (12)	0.0295 (6)*	
C12	1.0036 (2)	0.21518 (19)	0.13421 (13)	0.0265 (6)	
H12	1.0548 (2)	0.16992 (19)	0.12624 (13)	0.0318 (7)*	
C13	1.0146 (2)	0.3047 (2)	0.11526 (14)	0.0288 (6)	
H13	1.0736 (2)	0.3207 (2)	0.09430 (14)	0.0345 (7)*	
C14	0.9401 (2)	0.37174 (18)	0.12653 (12)	0.0225 (5)	
H14	0.9483 (2)	0.43300 (18)	0.11321 (12)	0.0270 (6)*	
C15	0.80299 (18)	0.54531 (16)	0.15972 (11)	0.0154 (4)	
C16	0.8012 (2)	0.57116 (17)	0.09857 (11)	0.0191 (5)	
H16	0.7725 (2)	0.53029 (17)	0.06704 (11)	0.0230 (6)*	
C17	0.8409 (2)	0.65579 (18)	0.08390 (12)	0.0239 (5)	
H17	0.8407 (2)	0.67245 (18)	0.04241 (12)	0.0287 (6)*	
C18	0.8810 (2)	0.71640 (18)	0.12988 (13)	0.0243 (5)	
H18	0.9091 (2)	0.77415 (18)	0.11975 (13)	0.0292 (6)*	
C19	0.8802 (2)	0.69309 (17)	0.19026 (12)	0.0230 (5)	
H19	0.9061 (2)	0.73538 (17)	0.22151 (12)	0.0276 (6)*	
C20	0.84132 (19)	0.60752 (16)	0.20539 (11)	0.0184 (5)	
H20	0.84098 (19)	0.59157 (16)	0.24696 (11)	0.0221 (6)*	
C21	0.63591 (19)	0.41706 (18)	0.12270 (11)	0.0184 (5)	
C22	0.6201 (2)	0.3401 (2)	0.08494 (11)	0.0249 (6)	
H22	0.6749 (2)	0.2945 (2)	0.08626 (11)	0.0299 (7)*	
C23	0.5236 (2)	0.3301 (3)	0.04523 (13)	0.0368 (7)	
H23	0.5130 (2)	0.2778 (3)	0.01956 (13)	0.0442 (9)*	
C24	0.4443 (2)	0.3958 (3)	0.04325 (14)	0.0414 (8)	
H24	0.3790 (2)	0.3887 (3)	0.01610 (14)	0.0496 (10)*	
C25	0.4588 (2)	0.4726 (2)	0.08064 (15)	0.0381 (7)	
H25	0.4034 (2)	0.5178 (2)	0.07906 (15)	0.0457 (9)*	
C26	0.5542 (2)	0.4834 (2)	0.12030 (13)	0.0271 (6)	
H26	0.5641 (2)	0.5360 (2)	0.14585 (13)	0.0325 (7)*	
C27	0.5847 (2)	0.34407 (17)	0.39776 (11)	0.0188 (5)	
C28	0.5216 (2)	0.26974 (17)	0.37417 (11)	0.0200 (5)	
H28	0.5532 (2)	0.22330 (17)	0.35212 (11)	0.0240 (6)*	
C29	0.4137 (2)	0.26292 (18)	0.38253 (12)	0.0236 (5)	
H29	0.3715 (2)	0.21221 (18)	0.36610 (12)	0.0283 (6)*	
C30	0.3671 (2)	0.3304 (2)	0.41501 (14)	0.0317 (6)	
H30	0.2933 (2)	0.3254 (2)	0.42116 (14)	0.0380 (8)*	
C31	0.4281 (2)	0.4047 (2)	0.43836 (14)	0.0356 (7)	
H31	0.3959 (2)	0.4510 (2)	0.46022 (14)	0.0427 (9)*	
C32	0.5367 (2)	0.4118 (2)	0.42990 (13)	0.0280 (6)	
H32	0.5784 (2)	0.4629 (2)	0.44608 (13)	0.0336 (7)*	
C33	0.78511 (19)	0.43655 (16)	0.43603 (10)	0.0159 (4)	
C34	0.7724 (2)	0.52981 (17)	0.42266 (11)	0.0200 (5)	
H34	0.7352 (2)	0.54844 (17)	0.38434 (11)	0.0240 (6)*	
C35	0.8135 (2)	0.59552 (18)	0.46483 (12)	0.0236 (5)	
H35	0.8030 (2)	0.65889 (18)	0.45582 (12)	0.0283 (6)*	
C36	0.8697 (2)	0.56859 (19)	0.51989 (12)	0.0267 (6)	
H36	0.8993 (2)	0.61353 (19)	0.54848 (12)	0.0321 (7)*	
C37	0.8832 (2)	0.4761 (2)	0.53369 (12)	0.0307 (6)	
H37	0.9216 (2)	0.4579 (2)	0.57181 (12)	0.0369 (7)*	
C38	0.8410 (2)	0.41012 (19)	0.49217 (12)	0.0246 (5)	
H38	0.8501 (2)	0.34685 (19)	0.50189 (12)	0.0296 (6)*	
C39	0.77923 (19)	0.24282 (16)	0.39698 (11)	0.0177 (5)	
C40	0.8394 (2)	0.19755 (17)	0.35779 (12)	0.0209 (5)	
H40	0.8537 (2)	0.22686 (17)	0.32139 (12)	0.0251 (6)*	
C41	0.8791 (2)	0.10941 (19)	0.37135 (14)	0.0276 (6)	
H41	0.9192 (2)	0.07855 (19)	0.34395 (14)	0.0331 (7)*	
C42	0.8598 (2)	0.06684 (19)	0.42476 (15)	0.0321 (6)	
H42	0.8878 (2)	0.00718 (19)	0.43443 (15)	0.0385 (8)*	
C43	0.7999 (2)	0.1115 (2)	0.46389 (14)	0.0326 (6)	
H43	0.7870 (2)	0.0823 (2)	0.50059 (14)	0.0392 (8)*	
C44	0.7584 (2)	0.19844 (19)	0.45023 (12)	0.0254 (5)	
H44	0.7158 (2)	0.22789 (19)	0.47703 (12)	0.0304 (7)*	
F2	0.3380 (3)	0.16952 (18)	0.21762 (13)	0.0886 (9)	
F3	0.2740 (2)	0.2539 (2)	0.14237 (17)	0.1024 (10)	

### (1) Atomic displacement parameters (Å^2^)

**Table:**

	*U*^11^	*U*^22^	*U*^33^	*U*^12^	*U*^13^	*U*^23^
I1	0.01334 (8)	0.01817 (8)	0.01806 (8)	−0.00094 (5)	−0.00148 (6)	0.00035 (5)
Pd1	0.01028 (8)	0.01240 (9)	0.01125 (8)	−0.00042 (6)	0.00020 (6)	−0.00016 (6)
P1	0.0127 (3)	0.0130 (3)	0.0121 (3)	−0.0007 (2)	0.0007 (2)	0.0007 (2)
F1	0.0375 (10)	0.0327 (9)	0.0395 (10)	−0.0128 (8)	0.0073 (8)	−0.0185 (8)
C1	0.0162 (11)	0.0176 (11)	0.0134 (10)	−0.0013 (9)	0.0026 (8)	0.0015 (9)
P2	0.0138 (3)	0.0150 (3)	0.0121 (3)	−0.0013 (2)	0.0013 (2)	0.0001 (2)
C2	0.0149 (11)	0.0147 (11)	0.0193 (11)	−0.0008 (9)	0.0028 (9)	−0.0006 (9)
C3	0.0193 (12)	0.0200 (12)	0.0247 (13)	−0.0033 (9)	0.0012 (10)	−0.0026 (10)
F4	0.0491 (12)	0.0278 (10)	0.0807 (16)	0.0168 (9)	−0.0006 (11)	−0.0011 (10)
C4	0.0115 (11)	0.0273 (13)	0.0282 (13)	−0.0004 (10)	−0.0026 (9)	−0.0009 (11)
F5	0.0505 (12)	0.0528 (13)	0.0549 (13)	0.0235 (10)	0.0069 (10)	−0.0256 (10)
C5	0.0175 (12)	0.0212 (12)	0.0297 (13)	0.0040 (10)	0.0022 (10)	−0.0040 (10)
F6	0.0206 (9)	0.0466 (12)	0.118 (2)	0.0061 (8)	0.0127 (11)	−0.0308 (13)
C6	0.0164 (11)	0.0183 (11)	0.0226 (12)	−0.0004 (9)	−0.0018 (9)	−0.0008 (9)
C7	0.0173 (13)	0.0323 (15)	0.056 (2)	−0.0077 (11)	0.0044 (12)	−0.0179 (14)
C8	0.0214 (14)	0.0300 (15)	0.0549 (19)	0.0044 (12)	−0.0009 (13)	−0.0145 (14)
C9	0.0163 (11)	0.0146 (11)	0.0130 (10)	−0.0002 (8)	−0.0002 (8)	−0.0027 (8)
C10	0.0242 (12)	0.0162 (11)	0.0228 (12)	−0.0015 (9)	0.0074 (10)	0.0009 (9)
C11	0.0310 (14)	0.0145 (11)	0.0285 (13)	0.0012 (10)	0.0054 (11)	−0.0022 (10)
C12	0.0255 (13)	0.0224 (13)	0.0322 (14)	0.0075 (10)	0.0058 (11)	−0.0048 (11)
C13	0.0233 (13)	0.0286 (14)	0.0370 (15)	0.0028 (11)	0.0138 (11)	0.0031 (12)
C14	0.0222 (12)	0.0195 (12)	0.0264 (13)	−0.0014 (10)	0.0060 (10)	0.0025 (10)
C15	0.0140 (10)	0.0143 (10)	0.0177 (11)	0.0010 (8)	0.0016 (8)	0.0020 (9)
C16	0.0203 (12)	0.0192 (12)	0.0180 (12)	0.0008 (9)	0.0029 (9)	0.0008 (9)
C17	0.0227 (13)	0.0252 (13)	0.0238 (13)	0.0011 (10)	0.0027 (10)	0.0098 (10)
C18	0.0190 (12)	0.0173 (12)	0.0355 (14)	−0.0027 (9)	−0.0009 (10)	0.0100 (10)
C19	0.0226 (12)	0.0158 (12)	0.0287 (13)	−0.0012 (9)	−0.0037 (10)	0.0015 (10)
C20	0.0176 (11)	0.0171 (11)	0.0196 (12)	0.0013 (9)	−0.0007 (9)	0.0015 (9)
C21	0.0152 (11)	0.0268 (13)	0.0126 (11)	−0.0045 (9)	0.0000 (9)	0.0053 (9)
C22	0.0206 (12)	0.0377 (15)	0.0163 (12)	−0.0068 (11)	0.0017 (10)	−0.0037 (10)
C23	0.0300 (15)	0.059 (2)	0.0199 (13)	−0.0176 (14)	−0.0027 (11)	−0.0039 (13)
C24	0.0226 (14)	0.067 (2)	0.0299 (16)	−0.0144 (15)	−0.0121 (12)	0.0148 (15)
C25	0.0211 (14)	0.0486 (19)	0.0414 (17)	0.0008 (13)	−0.0070 (12)	0.0222 (15)
C26	0.0215 (13)	0.0285 (14)	0.0298 (14)	−0.0012 (11)	−0.0017 (11)	0.0101 (11)
C27	0.0184 (11)	0.0239 (12)	0.0145 (11)	−0.0022 (9)	0.0032 (9)	0.0022 (9)
C28	0.0209 (12)	0.0198 (12)	0.0199 (12)	−0.0005 (9)	0.0046 (9)	0.0003 (9)
C29	0.0210 (12)	0.0224 (13)	0.0275 (13)	−0.0054 (10)	0.0040 (10)	−0.0017 (10)
C30	0.0190 (13)	0.0437 (17)	0.0344 (15)	−0.0065 (12)	0.0109 (11)	−0.0095 (13)
C31	0.0247 (14)	0.0468 (18)	0.0376 (16)	−0.0048 (13)	0.0127 (12)	−0.0219 (14)
C32	0.0228 (13)	0.0355 (15)	0.0268 (14)	−0.0073 (11)	0.0070 (11)	−0.0145 (11)
C33	0.0148 (11)	0.0197 (11)	0.0134 (11)	−0.0023 (9)	0.0022 (8)	−0.0011 (9)
C34	0.0227 (12)	0.0203 (12)	0.0165 (11)	0.0001 (10)	0.0009 (9)	−0.0004 (9)
C35	0.0286 (14)	0.0191 (12)	0.0236 (13)	−0.0014 (10)	0.0050 (11)	−0.0045 (10)
C36	0.0275 (14)	0.0291 (14)	0.0224 (13)	−0.0012 (11)	−0.0010 (11)	−0.0145 (11)
C37	0.0361 (15)	0.0337 (15)	0.0188 (13)	0.0033 (12)	−0.0090 (11)	−0.0042 (11)
C38	0.0302 (14)	0.0227 (13)	0.0193 (12)	0.0027 (10)	−0.0029 (10)	−0.0004 (10)
C39	0.0150 (11)	0.0167 (11)	0.0202 (11)	−0.0036 (9)	−0.0015 (9)	0.0025 (9)
C40	0.0199 (12)	0.0196 (12)	0.0233 (12)	−0.0006 (9)	0.0032 (10)	0.0024 (10)
C41	0.0234 (13)	0.0211 (13)	0.0385 (16)	0.0040 (10)	0.0053 (11)	0.0020 (11)
C42	0.0280 (14)	0.0196 (13)	0.0476 (18)	0.0018 (11)	0.0007 (13)	0.0103 (12)
C43	0.0364 (16)	0.0272 (14)	0.0338 (16)	−0.0027 (12)	0.0031 (12)	0.0149 (12)
C44	0.0294 (14)	0.0246 (13)	0.0225 (13)	−0.0005 (11)	0.0049 (11)	0.0066 (10)
F2	0.132 (2)	0.0553 (14)	0.0952 (18)	−0.0607 (13)	0.0757 (15)	−0.0352 (12)
F3	0.0467 (13)	0.0751 (16)	0.166 (3)	0.0176 (11)	−0.0553 (13)	−0.0687 (15)

### (1) Geometric parameters (Å, º)

**Table:**

I1—Pd1	2.6887 (2)	C19—C20	1.394 (3)
Pd1—P1	2.3206 (6)	C20—H20	0.9500
Pd1—C1	2.019 (2)	C21—C22	1.397 (4)
Pd1—P2	2.3240 (6)	C21—C26	1.399 (4)
P1—C9	1.825 (2)	C22—H22	0.9500
P1—C15	1.820 (2)	C22—C23	1.397 (4)
P1—C21	1.827 (2)	C23—H23	0.9500
F1—C7	1.316 (3)	C23—C24	1.371 (5)
C1—C2	1.396 (3)	C24—H24	0.9500
C1—C6	1.389 (3)	C24—C25	1.390 (5)
P2—C27	1.825 (2)	C25—H25	0.9500
P2—C33	1.821 (2)	C25—C26	1.388 (4)
P2—C39	1.823 (2)	C26—H26	0.9500
C2—H2	0.9500	C27—C28	1.397 (3)
C2—C3	1.394 (3)	C27—C32	1.397 (4)
C3—C4	1.382 (4)	C28—H28	0.9500
C3—C7	1.494 (4)	C28—C29	1.385 (3)
F4—C8	1.351 (4)	C29—H29	0.9500
C4—H4	0.9500	C29—C30	1.390 (4)
C4—C5	1.380 (4)	C30—H30	0.9500
F5—C8	1.333 (4)	C30—C31	1.382 (4)
C5—C6	1.405 (3)	C31—H31	0.9500
C5—C8	1.498 (4)	C31—C32	1.394 (4)
F6—C8	1.324 (3)	C32—H32	0.9500
C6—H6	0.9500	C33—C34	1.395 (3)
C7—F2	1.330 (4)	C33—C38	1.395 (3)
C7—F3	1.321 (4)	C34—H34	0.9500
C9—C10	1.402 (3)	C34—C35	1.387 (3)
C9—C14	1.392 (3)	C35—H35	0.9500
C10—H10	0.9500	C35—C36	1.379 (4)
C10—C11	1.384 (4)	C36—H36	0.9500
C11—H11	0.9500	C36—C37	1.388 (4)
C11—C12	1.384 (4)	C37—H37	0.9500
C12—H12	0.9500	C37—C38	1.384 (4)
C12—C13	1.383 (4)	C38—H38	0.9500
C13—H13	0.9500	C39—C40	1.387 (3)
C13—C14	1.392 (4)	C39—C44	1.400 (3)
C14—H14	0.9500	C40—H40	0.9500
C15—C16	1.403 (3)	C40—C41	1.394 (4)
C15—C20	1.394 (3)	C41—H41	0.9500
C16—H16	0.9500	C41—C42	1.385 (4)
C16—C17	1.383 (3)	C42—H42	0.9500
C17—H17	0.9500	C42—C43	1.380 (4)
C17—C18	1.389 (4)	C43—H43	0.9500
C18—H18	0.9500	C43—C44	1.387 (4)
C18—C19	1.381 (4)	C44—H44	0.9500
C19—H19	0.9500		
			
P1—Pd1—I1	90.186 (15)	H19—C19—C18	119.98 (15)
C1—Pd1—I1	176.51 (7)	C20—C19—C18	120.0 (2)
C1—Pd1—P1	89.90 (7)	C20—C19—H19	119.98 (15)
P2—Pd1—I1	90.764 (15)	C19—C20—C15	120.2 (2)
P2—Pd1—P1	173.08 (2)	H20—C20—C15	119.90 (14)
P2—Pd1—C1	89.57 (7)	H20—C20—C19	119.90 (15)
C9—P1—Pd1	106.57 (8)	C22—C21—P1	122.8 (2)
C15—P1—Pd1	117.58 (8)	C26—C21—P1	117.9 (2)
C15—P1—C9	107.21 (11)	C26—C21—C22	119.3 (2)
C21—P1—Pd1	117.73 (8)	H22—C22—C21	119.98 (15)
C21—P1—C9	104.70 (11)	C23—C22—C21	120.0 (3)
C21—P1—C15	101.99 (11)	C23—C22—H22	120.0 (2)
C2—C1—Pd1	122.82 (17)	H23—C23—C22	120.0 (2)
C6—C1—Pd1	119.15 (17)	C24—C23—C22	120.1 (3)
C6—C1—C2	118.0 (2)	C24—C23—H23	119.96 (18)
C27—P2—Pd1	116.71 (8)	H24—C24—C23	119.72 (18)
C33—P2—Pd1	112.22 (8)	C25—C24—C23	120.6 (3)
C33—P2—C27	103.43 (11)	C25—C24—H24	119.72 (18)
C39—P2—Pd1	113.20 (8)	H25—C25—C24	120.02 (18)
C39—P2—C27	102.14 (11)	C26—C25—C24	120.0 (3)
C39—P2—C33	108.13 (11)	C26—C25—H25	120.0 (2)
H2—C2—C1	119.64 (14)	C25—C26—C21	120.1 (3)
C3—C2—C1	120.7 (2)	H26—C26—C21	119.95 (16)
C3—C2—H2	119.64 (14)	H26—C26—C25	119.9 (2)
C4—C3—C2	121.0 (2)	C28—C27—P2	119.18 (19)
C7—C3—C2	119.2 (2)	C32—C27—P2	121.75 (19)
C7—C3—C4	119.7 (2)	C32—C27—C28	118.7 (2)
H4—C4—C3	120.58 (15)	H28—C28—C27	119.58 (14)
C5—C4—C3	118.8 (2)	C29—C28—C27	120.8 (2)
C5—C4—H4	120.58 (15)	C29—C28—H28	119.58 (15)
C6—C5—C4	120.5 (2)	H29—C29—C28	120.06 (15)
C8—C5—C4	121.0 (2)	C30—C29—C28	119.9 (2)
C8—C5—C6	118.5 (2)	C30—C29—H29	120.06 (16)
C5—C6—C1	120.9 (2)	H30—C30—C29	119.96 (16)
H6—C6—C1	119.56 (14)	C31—C30—C29	120.1 (2)
H6—C6—C5	119.56 (15)	C31—C30—H30	119.96 (17)
C3—C7—F1	114.6 (2)	H31—C31—C30	119.93 (17)
F2—C7—F1	103.7 (3)	C32—C31—C30	120.1 (3)
F2—C7—C3	112.0 (3)	C32—C31—H31	119.93 (17)
F3—C7—F1	105.6 (3)	C31—C32—C27	120.3 (3)
F3—C7—C3	112.7 (3)	H32—C32—C27	119.84 (15)
F3—C7—F2	107.5 (3)	H32—C32—C31	119.84 (17)
F5—C8—F4	104.5 (2)	C34—C33—P2	116.86 (18)
C5—C8—F4	111.7 (3)	C38—C33—P2	123.97 (19)
C5—C8—F5	112.9 (3)	C38—C33—C34	119.1 (2)
F6—C8—F4	105.3 (3)	H34—C34—C33	119.72 (14)
F6—C8—F5	108.7 (3)	C35—C34—C33	120.6 (2)
F6—C8—C5	113.1 (2)	C35—C34—H34	119.72 (15)
C10—C9—P1	117.26 (18)	H35—C35—C34	120.11 (15)
C14—C9—P1	123.67 (18)	C36—C35—C34	119.8 (2)
C14—C9—C10	119.0 (2)	C36—C35—H35	120.11 (15)
H10—C10—C9	119.69 (14)	H36—C36—C35	119.89 (15)
C11—C10—C9	120.6 (2)	C37—C36—C35	120.2 (2)
C11—C10—H10	119.69 (15)	C37—C36—H36	119.89 (15)
H11—C11—C10	119.94 (15)	H37—C37—C36	119.85 (15)
C12—C11—C10	120.1 (2)	C38—C37—C36	120.3 (2)
C12—C11—H11	119.94 (15)	C38—C37—H37	119.85 (16)
H12—C12—C11	120.15 (15)	C37—C38—C33	120.0 (2)
C13—C12—C11	119.7 (2)	H38—C38—C33	120.00 (15)
C13—C12—H12	120.15 (16)	H38—C38—C37	120.00 (16)
H13—C13—C12	119.61 (16)	C40—C39—P2	121.38 (18)
C14—C13—C12	120.8 (2)	C44—C39—P2	119.6 (2)
C14—C13—H13	119.61 (16)	C44—C39—C40	119.0 (2)
C13—C14—C9	119.9 (2)	H40—C40—C39	119.72 (14)
H14—C14—C9	120.07 (14)	C41—C40—C39	120.6 (2)
H14—C14—C13	120.07 (16)	C41—C40—H40	119.72 (16)
C16—C15—P1	120.36 (18)	H41—C41—C40	120.00 (16)
C20—C15—P1	120.60 (18)	C42—C41—C40	120.0 (3)
C20—C15—C16	119.0 (2)	C42—C41—H41	120.00 (17)
H16—C16—C15	119.79 (14)	H42—C42—C41	120.16 (17)
C17—C16—C15	120.4 (2)	C43—C42—C41	119.7 (3)
C17—C16—H16	119.79 (15)	C43—C42—H42	120.16 (16)
H17—C17—C16	120.03 (15)	H43—C43—C42	119.63 (16)
C18—C17—C16	119.9 (2)	C44—C43—C42	120.7 (3)
C18—C17—H17	120.03 (15)	C44—C43—H43	119.63 (17)
H18—C18—C17	119.85 (15)	C43—C44—C39	120.0 (3)
C19—C18—C17	120.3 (2)	H44—C44—C39	120.00 (15)
C19—C18—H18	119.85 (15)	H44—C44—C43	120.00 (17)
			
Pd1—C1—C2—C3	−179.72 (19)	C4—C5—C8—F5	136.1 (3)
Pd1—C1—C6—C5	179.74 (19)	C4—C5—C8—F6	12.1 (3)
P1—C9—C10—C11	−176.36 (19)	F5—C8—C5—C6	−46.4 (3)
P1—C9—C14—C13	175.9 (2)	C9—C10—C11—C12	0.2 (3)
P1—C15—C16—C17	−177.40 (19)	C9—C14—C13—C12	0.2 (3)
P1—C15—C20—C19	178.06 (19)	C10—C11—C12—C13	−0.1 (3)
P1—C21—C22—C23	176.3 (2)	C11—C12—C13—C14	−0.1 (3)
P1—C21—C26—C25	−176.5 (2)	C15—C16—C17—C18	−1.2 (3)
F1—C7—C3—C2	−26.8 (3)	C15—C20—C19—C18	−0.2 (3)
F1—C7—C3—C4	155.1 (3)	C16—C17—C18—C19	−0.9 (3)
C1—C2—C3—C4	0.2 (3)	C17—C18—C19—C20	1.5 (3)
C1—C2—C3—C7	−177.8 (3)	C21—C22—C23—C24	0.0 (3)
C1—C6—C5—C4	−0.2 (3)	C21—C26—C25—C24	0.0 (3)
C1—C6—C5—C8	−177.6 (3)	C22—C23—C24—C25	−0.1 (3)
P2—C27—C28—C29	−173.66 (19)	C23—C24—C25—C26	0.2 (4)
P2—C27—C32—C31	173.6 (2)	C27—C28—C29—C30	−0.3 (3)
P2—C33—C34—C35	177.09 (19)	C27—C32—C31—C30	0.1 (4)
P2—C33—C38—C37	−177.9 (2)	C28—C29—C30—C31	0.7 (3)
P2—C39—C40—C41	−177.7 (2)	C29—C30—C31—C32	−0.6 (4)
P2—C39—C44—C43	178.9 (2)	C33—C34—C35—C36	1.5 (3)
C2—C3—C4—C5	0.1 (3)	C33—C38—C37—C36	0.3 (3)
C2—C3—C7—F2	91.0 (3)	C34—C35—C36—C37	−1.4 (3)
C2—C3—C7—F3	−147.7 (3)	C35—C36—C37—C38	0.4 (3)
C3—C4—C5—C6	−0.1 (3)	C39—C40—C41—C42	−1.0 (3)
C3—C4—C5—C8	177.3 (3)	C39—C44—C43—C42	−1.5 (3)
F4—C8—C5—C4	−106.4 (3)	C40—C41—C42—C43	1.0 (3)
F4—C8—C5—C6	71.0 (3)	C41—C42—C43—C44	0.2 (3)

### (3,5-Dinitrophenyl)iodidobis(triphenylphosphane)palladium(II) ethyl acetate monosolvate (2) Crystal data

**Table:**

[Pd(C_6_H_3_N_2_O4)I(C_18_H_15_P)_2_]·C_4_H_8_O_2_	*F*(000) = 2028.373
*M**_r_* = 1013.12	*D*_x_ = 1.595 Mg m^−^^3^
Monoclinic, *P*2_1_/*c*	Mo *K*α radiation, λ = 0.71073 Å
*a* = 11.7692 (7) Å	Cell parameters from 6764 reflections
*b* = 34.870 (2) Å	θ = 2.3–24.5°
*c* = 10.4381 (6) Å	µ = 1.30 mm^−^^1^
β = 99.920 (1)°	*T* = 100 K
*V* = 4219.7 (4) Å^3^	Block, clear yellow
*Z* = 4	0.4 × 0.25 × 0.1 mm

### (3,5-Dinitrophenyl)iodidobis(triphenylphosphane)palladium(II) ethyl acetate monosolvate (2) Data collection

**Table:**

Bruker APEXI CCD diffractometer	7734 reflections with *I*≥ 2u(*I*)
φ and ω scans	*R*_int_ = 0.073
Absorption correction: multi-scan (SADABS; Krause *et al.*, 2015)	θ_max_ = 27.1°, θ_min_ = 1.2°
*T*_min_ = 0.663, *T*_max_ = 0.746	*h* = −15→15
53507 measured reflections	*k* = −44→44
9320 independent reflections	*l* = −13→13

### (3,5-Dinitrophenyl)iodidobis(triphenylphosphane)palladium(II) ethyl acetate monosolvate (2) Refinement

**Table:**

Refinement on *F*^2^	78 constraints
Least-squares matrix: full	Primary atom site location: iterative
*R*[*F*^2^ > 2σ(*F*^2^)] = 0.045	H-atom parameters constrained
*wR*(*F*^2^) = 0.091	*w* = 1/[σ^2^(*F*_o_^2^) + (0.0221*P*)^2^ + 15.765*P*] where *P* = (*F*_o_^2^ + 2*F*_c_^2^)/3
*S* = 1.06	(Δ/σ)_max_ = 0.001
9320 reflections	Δρ_max_ = 1.50 e Å^−^^3^
519 parameters	Δρ_min_ = −1.32 e Å^−^^3^
0 restraints	

### (3,5-Dinitrophenyl)iodidobis(triphenylphosphane)palladium(II) ethyl acetate monosolvate (2) Fractional atomic coordinates and isotropic or equivalent isotropic displacement parameters (Å^2^)

**Table:**

	*x*	*y*	*z*	*U*_iso_*/*U*_eq_	
I1	0.40258 (2)	0.413322 (8)	0.22478 (3)	0.01801 (8)	
Pd1	0.59200 (2)	0.373950 (8)	0.32437 (3)	0.01022 (7)	
P1	0.70748 (9)	0.42072 (3)	0.25352 (10)	0.0120 (2)	
O1	0.8235 (3)	0.35967 (10)	0.8178 (3)	0.0306 (8)	
N1	0.8900 (3)	0.34076 (11)	0.7649 (4)	0.0226 (8)	
C1	0.7341 (3)	0.34829 (10)	0.4201 (4)	0.0068 (5)	
P2	0.48101 (8)	0.32165 (3)	0.36841 (10)	0.0103 (2)	
O2	0.9761 (3)	0.32500 (10)	0.8243 (3)	0.0342 (9)	
N2	0.9728 (3)	0.28312 (10)	0.3628 (4)	0.0194 (8)	
C2	0.7994 (3)	0.32503 (10)	0.3576 (4)	0.0068 (5)	
H2	0.7792 (3)	0.32071 (10)	0.2666 (4)	0.0082 (6)*	
O3	1.0625 (3)	0.27056 (10)	0.4255 (3)	0.0326 (8)	
C3	0.8990 (3)	0.30722 (11)	0.4311 (4)	0.0160 (9)	
O4	0.9426 (3)	0.27755 (10)	0.2460 (3)	0.0275 (8)	
C4	0.9321 (4)	0.31185 (12)	0.5643 (4)	0.0185 (9)	
H4	0.9980 (4)	0.29947 (12)	0.6119 (4)	0.0222 (11)*	
O5	1.0288 (3)	0.40709 (10)	0.7260 (3)	0.0350 (9)	
C5	0.8628 (3)	0.33565 (12)	0.6230 (4)	0.0164 (9)	
O6	1.0202 (3)	0.45798 (9)	0.8545 (3)	0.0308 (8)	
C6	0.7664 (3)	0.35435 (11)	0.5547 (4)	0.0151 (8)	
H6	0.7227 (3)	0.37115 (11)	0.5989 (4)	0.0182 (10)*	
C7	0.3783 (3)	0.31053 (11)	0.2213 (4)	0.0111 (8)	
C8	0.4196 (4)	0.31309 (11)	0.1038 (4)	0.0151 (8)	
H8	0.4986 (4)	0.31887 (11)	0.1042 (4)	0.0181 (10)*	
C9	0.3462 (4)	0.30727 (11)	−0.0130 (4)	0.0163 (9)	
H9	0.3747 (4)	0.30899 (11)	−0.0926 (4)	0.0196 (10)*	
C10	0.2316 (4)	0.29895 (11)	−0.0138 (4)	0.0160 (8)	
H10	0.1809 (4)	0.29528 (11)	−0.0940 (4)	0.0192 (10)*	
C11	0.1905 (3)	0.29594 (12)	0.1024 (4)	0.0167 (9)	
H11	0.1115 (3)	0.29016 (12)	0.1015 (4)	0.0200 (10)*	
C12	0.2641 (3)	0.30131 (11)	0.2199 (4)	0.0141 (8)	
H12	0.2359 (3)	0.29865 (11)	0.2994 (4)	0.0169 (10)*	
C13	0.5555 (3)	0.27629 (11)	0.4127 (4)	0.0115 (8)	
C14	0.5513 (4)	0.24542 (12)	0.3294 (4)	0.0197 (9)	
H14	0.5016 (4)	0.24607 (12)	0.2474 (4)	0.0237 (11)*	
C15	0.6200 (4)	0.21314 (13)	0.3653 (5)	0.0273 (11)	
H15	0.6160 (4)	0.19186 (13)	0.3078 (5)	0.0327 (13)*	
C16	0.6932 (4)	0.21195 (13)	0.4828 (5)	0.0263 (11)	
H16	0.7417 (4)	0.19033 (13)	0.5052 (5)	0.0316 (13)*	
C17	0.6961 (4)	0.24229 (13)	0.5682 (5)	0.0225 (10)	
H17	0.7452 (4)	0.24129 (13)	0.6505 (5)	0.0271 (12)*	
C18	0.6274 (3)	0.27425 (12)	0.5341 (4)	0.0163 (9)	
H18	0.6292 (3)	0.29493 (12)	0.5936 (4)	0.0196 (10)*	
C19	0.3964 (3)	0.32934 (11)	0.4967 (4)	0.0131 (8)	
C20	0.3388 (3)	0.29901 (12)	0.5443 (4)	0.0143 (8)	
H20	0.3499 (3)	0.27357 (12)	0.5163 (4)	0.0172 (10)*	
C21	0.2655 (4)	0.30588 (12)	0.6323 (4)	0.0164 (9)	
H21	0.2252 (4)	0.28517 (12)	0.6631 (4)	0.0196 (10)*	
C22	0.2506 (4)	0.34279 (12)	0.6754 (4)	0.0170 (9)	
H22	0.1986 (4)	0.34754 (12)	0.7339 (4)	0.0204 (10)*	
C23	0.3118 (4)	0.37266 (12)	0.6330 (4)	0.0190 (9)	
H23	0.3041 (4)	0.39784 (12)	0.6652 (4)	0.0228 (11)*	
C24	0.3844 (4)	0.36609 (12)	0.5435 (4)	0.0153 (8)	
H24	0.4258 (4)	0.38678 (12)	0.5144 (4)	0.0184 (10)*	
C25	0.6540 (3)	0.43208 (12)	0.0840 (4)	0.0149 (8)	
C26	0.6448 (4)	0.46947 (13)	0.0354 (5)	0.0263 (11)	
H26	0.6707 (4)	0.49048 (13)	0.0908 (5)	0.0315 (13)*	
C27	0.5975 (5)	0.47580 (15)	−0.0946 (5)	0.0368 (13)	
H27	0.5892 (5)	0.50128 (15)	−0.1271 (5)	0.0441 (15)*	
C28	0.5626 (5)	0.44535 (16)	−0.1769 (5)	0.0358 (13)	
H28	0.5319 (5)	0.44992 (16)	−0.2658 (5)	0.0429 (15)*	
C29	0.5723 (4)	0.40866 (15)	−0.1301 (5)	0.0307 (11)	
H29	0.5479 (4)	0.38774 (15)	−0.1865 (5)	0.0368 (14)*	
C30	0.6174 (4)	0.40199 (13)	−0.0007 (4)	0.0234 (10)	
H30	0.6236 (4)	0.37640 (13)	0.0309 (4)	0.0281 (12)*	
C31	0.8580 (3)	0.40728 (11)	0.2589 (4)	0.0152 (8)	
C32	0.8967 (4)	0.39233 (12)	0.1503 (4)	0.0214 (9)	
H32	0.8470 (4)	0.39190 (12)	0.0683 (4)	0.0257 (11)*	
C33	1.0091 (4)	0.37794 (13)	0.1626 (5)	0.0288 (11)	
H33	1.0355 (4)	0.36731 (13)	0.0892 (5)	0.0346 (13)*	
C34	1.0817 (4)	0.37921 (13)	0.2816 (5)	0.0297 (12)	
H34	1.1586 (4)	0.37001 (13)	0.2893 (5)	0.0356 (14)*	
C35	1.0434 (4)	0.39364 (12)	0.3885 (5)	0.0237 (10)	
H35	1.0934 (4)	0.39384 (12)	0.4703 (5)	0.0284 (12)*	
C36	0.9326 (3)	0.40795 (11)	0.3786 (4)	0.0178 (9)	
H36	0.9072 (3)	0.41822 (11)	0.4531 (4)	0.0213 (11)*	
C37	0.7186 (3)	0.46461 (11)	0.3478 (4)	0.0136 (8)	
C38	0.8032 (4)	0.49187 (12)	0.3373 (4)	0.0208 (9)	
H38	0.8537 (4)	0.48804 (12)	0.2766 (4)	0.0249 (11)*	
C39	0.8145 (4)	0.52443 (12)	0.4142 (5)	0.0231 (10)	
H39	0.8723 (4)	0.54284 (12)	0.4059 (5)	0.0278 (12)*	
C40	0.7413 (4)	0.53017 (12)	0.5037 (4)	0.0210 (9)	
H40	0.7491 (4)	0.55245 (12)	0.5569 (4)	0.0252 (11)*	
C41	0.6573 (4)	0.50349 (12)	0.5150 (4)	0.0209 (9)	
H41	0.6073 (4)	0.50740 (12)	0.5762 (4)	0.0251 (11)*	
C42	0.6453 (4)	0.47077 (12)	0.4371 (4)	0.0174 (9)	
H42	0.5868 (4)	0.45259 (12)	0.4451 (4)	0.0209 (11)*	
C43	1.0684 (4)	0.42566 (13)	0.8203 (5)	0.0257 (10)	
C44	1.1783 (5)	0.41741 (15)	0.9107 (5)	0.0362 (13)	
H44a	1.1898 (16)	0.38961 (15)	0.918 (3)	0.0543 (19)*	
H44b	1.2429 (6)	0.4291 (9)	0.8769 (18)	0.0543 (19)*	
H44c	1.1744 (13)	0.4281 (9)	0.9967 (10)	0.0543 (19)*	
C45	0.9110 (4)	0.46948 (16)	0.7770 (5)	0.0365 (13)	
H45a	0.9186 (4)	0.47228 (16)	0.6845 (5)	0.0437 (15)*	
H45b	0.8507 (4)	0.45013 (16)	0.7835 (5)	0.0437 (15)*	
C46	0.8805 (5)	0.50695 (16)	0.8309 (5)	0.0409 (14)	
H46a	0.877 (3)	0.5039 (3)	0.9234 (10)	0.061 (2)*	
H46b	0.9395 (18)	0.5260 (3)	0.821 (3)	0.061 (2)*	
H46c	0.8055 (16)	0.5155 (6)	0.784 (3)	0.061 (2)*	

### (3,5-Dinitrophenyl)iodidobis(triphenylphosphane)palladium(II) ethyl acetate monosolvate (2) Atomic displacement parameters (Å^2^)

**Table:**

	*U*^11^	*U*^22^	*U*^33^	*U*^12^	*U*^13^	*U*^23^
I1	0.01500 (14)	0.01557 (14)	0.02268 (15)	0.00231 (11)	0.00105 (10)	0.00267 (11)
Pd1	0.00825 (14)	0.00941 (14)	0.01207 (15)	−0.00010 (11)	−0.00089 (11)	0.00154 (11)
P1	0.0113 (5)	0.0088 (5)	0.0158 (5)	−0.0002 (4)	0.0019 (4)	0.0001 (4)
O1	0.0281 (19)	0.047 (2)	0.0155 (17)	0.0058 (16)	0.0005 (14)	−0.0044 (15)
N1	0.020 (2)	0.027 (2)	0.0188 (19)	−0.0003 (16)	−0.0031 (16)	0.0015 (16)
C1	0.0058 (12)	0.0039 (12)	0.0104 (13)	−0.0023 (9)	0.0008 (10)	0.0019 (10)
P2	0.0100 (5)	0.0102 (5)	0.0101 (5)	−0.0001 (4)	−0.0002 (4)	0.0006 (4)
O2	0.034 (2)	0.044 (2)	0.0192 (17)	0.0143 (17)	−0.0108 (15)	0.0028 (15)
N2	0.0151 (18)	0.0134 (18)	0.028 (2)	0.0019 (14)	0.0005 (16)	−0.0032 (15)
C2	0.0058 (12)	0.0039 (12)	0.0104 (13)	−0.0023 (9)	0.0008 (10)	0.0019 (10)
O3	0.0234 (18)	0.037 (2)	0.034 (2)	0.0189 (15)	−0.0062 (15)	−0.0023 (16)
C3	0.015 (2)	0.013 (2)	0.020 (2)	−0.0004 (16)	0.0032 (17)	−0.0010 (16)
O4	0.0236 (17)	0.036 (2)	0.0228 (18)	0.0064 (14)	0.0023 (14)	−0.0100 (14)
C4	0.015 (2)	0.015 (2)	0.023 (2)	−0.0002 (16)	−0.0049 (17)	0.0038 (17)
O5	0.044 (2)	0.0292 (19)	0.032 (2)	−0.0066 (16)	0.0075 (17)	−0.0121 (16)
C5	0.016 (2)	0.017 (2)	0.015 (2)	−0.0017 (16)	−0.0025 (16)	0.0019 (16)
O6	0.034 (2)	0.0272 (18)	0.0275 (18)	0.0076 (15)	−0.0032 (15)	−0.0088 (14)
C6	0.015 (2)	0.014 (2)	0.015 (2)	−0.0017 (16)	−0.0003 (16)	−0.0005 (16)
C7	0.0125 (19)	0.0097 (18)	0.0111 (19)	0.0025 (15)	0.0016 (15)	−0.0004 (15)
C8	0.014 (2)	0.016 (2)	0.015 (2)	−0.0013 (16)	0.0035 (16)	0.0021 (16)
C9	0.022 (2)	0.013 (2)	0.014 (2)	0.0009 (16)	0.0018 (17)	−0.0001 (16)
C10	0.019 (2)	0.014 (2)	0.013 (2)	0.0020 (16)	−0.0024 (16)	−0.0005 (16)
C11	0.012 (2)	0.015 (2)	0.022 (2)	−0.0003 (16)	−0.0014 (17)	−0.0019 (17)
C12	0.0120 (19)	0.014 (2)	0.016 (2)	−0.0011 (15)	0.0025 (16)	−0.0002 (16)
C13	0.0119 (19)	0.0123 (19)	0.0112 (19)	−0.0009 (15)	0.0042 (15)	0.0026 (15)
C14	0.022 (2)	0.021 (2)	0.017 (2)	0.0064 (18)	0.0068 (18)	0.0031 (18)
C15	0.040 (3)	0.019 (2)	0.024 (2)	0.009 (2)	0.010 (2)	−0.0001 (19)
C16	0.026 (2)	0.023 (2)	0.034 (3)	0.014 (2)	0.016 (2)	0.015 (2)
C17	0.018 (2)	0.022 (2)	0.026 (2)	0.0010 (18)	0.0012 (19)	0.0122 (19)
C18	0.013 (2)	0.017 (2)	0.019 (2)	−0.0033 (16)	0.0032 (17)	0.0036 (17)
C19	0.0113 (19)	0.014 (2)	0.0123 (19)	0.0003 (15)	−0.0031 (15)	0.0005 (15)
C20	0.016 (2)	0.013 (2)	0.014 (2)	−0.0004 (16)	0.0001 (16)	−0.0002 (16)
C21	0.019 (2)	0.015 (2)	0.015 (2)	−0.0003 (16)	0.0007 (17)	0.0027 (16)
C22	0.017 (2)	0.023 (2)	0.011 (2)	0.0039 (17)	0.0027 (16)	−0.0006 (17)
C23	0.026 (2)	0.015 (2)	0.016 (2)	0.0054 (18)	0.0026 (18)	−0.0033 (17)
C24	0.018 (2)	0.014 (2)	0.013 (2)	−0.0018 (16)	0.0031 (16)	−0.0011 (16)
C25	0.0118 (19)	0.015 (2)	0.019 (2)	−0.0007 (16)	0.0053 (16)	0.0041 (16)
C26	0.036 (3)	0.015 (2)	0.028 (3)	−0.001 (2)	0.004 (2)	0.0069 (19)
C27	0.049 (3)	0.030 (3)	0.031 (3)	0.004 (2)	0.008 (3)	0.017 (2)
C28	0.039 (3)	0.048 (3)	0.018 (2)	0.005 (3)	0.001 (2)	0.013 (2)
C29	0.035 (3)	0.034 (3)	0.020 (2)	0.001 (2)	−0.003 (2)	0.002 (2)
C30	0.027 (2)	0.018 (2)	0.024 (2)	0.0008 (19)	0.0031 (19)	0.0020 (18)
C31	0.0126 (19)	0.0070 (19)	0.026 (2)	−0.0030 (15)	0.0033 (17)	0.0012 (16)
C32	0.021 (2)	0.019 (2)	0.026 (2)	−0.0007 (18)	0.0076 (19)	−0.0015 (18)
C33	0.025 (3)	0.024 (2)	0.042 (3)	0.000 (2)	0.019 (2)	−0.006 (2)
C34	0.015 (2)	0.015 (2)	0.059 (3)	−0.0022 (18)	0.006 (2)	−0.006 (2)
C35	0.014 (2)	0.013 (2)	0.041 (3)	−0.0042 (17)	−0.001 (2)	−0.003 (2)
C36	0.014 (2)	0.012 (2)	0.027 (2)	−0.0035 (16)	0.0013 (17)	−0.0033 (17)
C37	0.0128 (19)	0.0112 (19)	0.017 (2)	0.0021 (15)	0.0020 (16)	0.0015 (16)
C38	0.022 (2)	0.016 (2)	0.026 (2)	−0.0006 (18)	0.0104 (19)	−0.0027 (18)
C39	0.022 (2)	0.015 (2)	0.034 (3)	−0.0074 (18)	0.009 (2)	−0.0036 (19)
C40	0.026 (2)	0.013 (2)	0.024 (2)	0.0003 (18)	0.0015 (19)	−0.0040 (17)
C41	0.016 (2)	0.022 (2)	0.025 (2)	0.0007 (18)	0.0053 (18)	−0.0032 (19)
C42	0.016 (2)	0.015 (2)	0.022 (2)	−0.0016 (16)	0.0048 (17)	−0.0024 (17)
C43	0.035 (3)	0.022 (2)	0.022 (2)	−0.002 (2)	0.009 (2)	−0.0010 (19)
C44	0.047 (3)	0.035 (3)	0.025 (3)	0.016 (3)	0.002 (2)	−0.007 (2)
C45	0.030 (3)	0.042 (3)	0.034 (3)	0.006 (2)	−0.005 (2)	−0.007 (2)
C46	0.036 (3)	0.043 (3)	0.039 (3)	0.014 (3)	−0.007 (2)	−0.013 (3)

### (3,5-Dinitrophenyl)iodidobis(triphenylphosphane)palladium(II) ethyl acetate monosolvate (2) Geometric parameters (Å, º)

**Table:**

I1—Pd1	2.6715 (4)	C20—H20	0.9500
Pd1—P1	2.3240 (10)	C20—C21	1.385 (6)
Pd1—C1	2.005 (4)	C21—H21	0.9500
Pd1—P2	2.3347 (10)	C21—C22	1.385 (6)
P1—C25	1.816 (4)	C22—H22	0.9500
P1—C31	1.824 (4)	C22—C23	1.382 (6)
P1—C37	1.812 (4)	C23—H23	0.9500
O1—N1	1.225 (5)	C23—C24	1.389 (6)
N1—O2	1.223 (5)	C24—H24	0.9500
N1—C5	1.471 (5)	C25—C26	1.396 (6)
C1—C2	1.360 (5)	C25—C30	1.392 (6)
C1—C6	1.407 (5)	C26—H26	0.9500
P2—C7	1.825 (4)	C26—C27	1.393 (7)
P2—C13	1.828 (4)	C27—H27	0.9500
P2—C19	1.821 (4)	C27—C28	1.383 (8)
N2—O3	1.224 (4)	C28—H28	0.9500
N2—C3	1.477 (5)	C28—C29	1.367 (7)
N2—O4	1.226 (5)	C29—H29	0.9500
C2—H2	0.9500	C29—C30	1.383 (6)
C2—C3	1.428 (5)	C30—H30	0.9500
C3—C4	1.387 (6)	C31—C32	1.394 (6)
C4—H4	0.9500	C31—C36	1.398 (6)
C4—C5	1.379 (6)	C32—H32	0.9500
O5—C43	1.203 (5)	C32—C33	1.400 (6)
C5—C6	1.393 (5)	C33—H33	0.9500
O6—C43	1.337 (5)	C33—C34	1.382 (7)
O6—C45	1.452 (6)	C34—H34	0.9500
C6—H6	0.9500	C34—C35	1.369 (7)
C7—C8	1.398 (5)	C35—H35	0.9500
C7—C12	1.380 (5)	C35—C36	1.383 (6)
C8—H8	0.9500	C36—H36	0.9500
C8—C9	1.382 (6)	C37—C38	1.394 (6)
C9—H9	0.9500	C37—C42	1.393 (6)
C9—C10	1.378 (6)	C38—H38	0.9500
C10—H10	0.9500	C38—C39	1.384 (6)
C10—C11	1.385 (6)	C39—H39	0.9500
C11—H11	0.9500	C39—C40	1.391 (6)
C11—C12	1.387 (6)	C40—H40	0.9500
C12—H12	0.9500	C40—C41	1.377 (6)
C13—C14	1.379 (6)	C41—H41	0.9500
C13—C18	1.399 (5)	C41—C42	1.394 (6)
C14—H14	0.9500	C42—H42	0.9500
C14—C15	1.399 (6)	C43—C44	1.492 (7)
C15—H15	0.9500	C44—H44a	0.9800
C15—C16	1.373 (7)	C44—H44b	0.9800
C16—H16	0.9500	C44—H44c	0.9800
C16—C17	1.380 (7)	C45—H45a	0.9900
C17—H17	0.9500	C45—H45b	0.9900
C17—C18	1.387 (6)	C45—C46	1.490 (7)
C18—H18	0.9500	C46—H46a	0.9800
C19—C20	1.393 (5)	C46—H46b	0.9800
C19—C24	1.387 (5)	C46—H46c	0.9800
			
P1—Pd1—I1	90.58 (3)	C22—C21—C20	120.3 (4)
C1—Pd1—I1	172.63 (10)	C22—C21—H21	119.8 (2)
C1—Pd1—P1	89.35 (10)	H22—C22—C21	120.2 (2)
P2—Pd1—I1	91.17 (3)	C23—C22—C21	119.6 (4)
P2—Pd1—P1	171.36 (4)	C23—C22—H22	120.2 (2)
P2—Pd1—C1	90.00 (10)	H23—C23—C22	119.8 (2)
C25—P1—Pd1	109.84 (13)	C24—C23—C22	120.4 (4)
C31—P1—Pd1	115.34 (13)	C24—C23—H23	119.8 (2)
C31—P1—C25	104.95 (19)	C23—C24—C19	120.1 (4)
C37—P1—Pd1	114.31 (14)	H24—C24—C19	120.0 (2)
C37—P1—C25	108.97 (19)	H24—C24—C23	120.0 (2)
C37—P1—C31	102.79 (18)	C26—C25—P1	123.3 (3)
O2—N1—O1	123.4 (4)	C30—C25—P1	118.2 (3)
C5—N1—O1	118.3 (3)	C30—C25—C26	118.5 (4)
C5—N1—O2	118.3 (4)	H26—C26—C25	120.2 (3)
C2—C1—Pd1	121.3 (3)	C27—C26—C25	119.7 (4)
C6—C1—Pd1	119.3 (3)	C27—C26—H26	120.2 (3)
C6—C1—C2	119.4 (3)	H27—C27—C26	119.7 (3)
C7—P2—Pd1	108.18 (13)	C28—C27—C26	120.6 (5)
C13—P2—Pd1	117.73 (13)	C28—C27—H27	119.7 (3)
C13—P2—C7	104.36 (17)	H28—C28—C27	120.0 (3)
C19—P2—Pd1	115.16 (13)	C29—C28—C27	119.9 (5)
C19—P2—C7	106.45 (18)	C29—C28—H28	120.0 (3)
C19—P2—C13	103.94 (18)	H29—C29—C28	120.0 (3)
C3—N2—O3	118.1 (4)	C30—C29—C28	120.0 (5)
O4—N2—O3	123.4 (4)	C30—C29—H29	120.0 (3)
O4—N2—C3	118.5 (3)	C29—C30—C25	121.2 (4)
H2—C2—C1	120.6 (2)	H30—C30—C25	119.4 (2)
C3—C2—C1	118.8 (3)	H30—C30—C29	119.4 (3)
C3—C2—H2	120.6 (2)	C32—C31—P1	121.5 (3)
C2—C3—N2	119.0 (4)	C36—C31—P1	118.7 (3)
C4—C3—N2	117.6 (4)	C36—C31—C32	119.4 (4)
C4—C3—C2	123.4 (4)	H32—C32—C31	120.2 (3)
H4—C4—C3	122.3 (2)	C33—C32—C31	119.7 (4)
C5—C4—C3	115.5 (4)	C33—C32—H32	120.2 (3)
C5—C4—H4	122.3 (2)	H33—C33—C32	120.0 (3)
C4—C5—N1	118.5 (4)	C34—C33—C32	119.9 (4)
C6—C5—N1	118.4 (4)	C34—C33—H33	120.0 (3)
C6—C5—C4	123.1 (4)	H34—C34—C33	119.8 (3)
C45—O6—C43	117.4 (4)	C35—C34—C33	120.3 (4)
C5—C6—C1	119.8 (4)	C35—C34—H34	119.8 (3)
H6—C6—C1	120.1 (2)	H35—C35—C34	119.7 (3)
H6—C6—C5	120.1 (2)	C36—C35—C34	120.7 (4)
C8—C7—P2	116.4 (3)	C36—C35—H35	119.7 (3)
C12—C7—P2	124.1 (3)	C35—C36—C31	120.0 (4)
C12—C7—C8	119.4 (4)	H36—C36—C31	120.0 (2)
H8—C8—C7	119.8 (2)	H36—C36—C35	120.0 (3)
C9—C8—C7	120.4 (4)	C38—C37—P1	121.3 (3)
C9—C8—H8	119.8 (2)	C42—C37—P1	119.9 (3)
H9—C9—C8	120.0 (2)	C42—C37—C38	118.7 (4)
C10—C9—C8	119.9 (4)	H38—C38—C37	119.6 (2)
C10—C9—H9	120.0 (2)	C39—C38—C37	120.8 (4)
H10—C10—C9	120.0 (2)	C39—C38—H38	119.6 (3)
C11—C10—C9	120.0 (4)	H39—C39—C38	120.0 (3)
C11—C10—H10	120.0 (2)	C40—C39—C38	119.9 (4)
H11—C11—C10	119.8 (2)	C40—C39—H39	120.0 (2)
C12—C11—C10	120.3 (4)	H40—C40—C39	120.1 (2)
C12—C11—H11	119.8 (2)	C41—C40—C39	119.8 (4)
C11—C12—C7	120.0 (4)	C41—C40—H40	120.1 (3)
H12—C12—C7	120.0 (2)	H41—C41—C40	119.8 (3)
H12—C12—C11	120.0 (2)	C42—C41—C40	120.4 (4)
C14—C13—P2	123.5 (3)	C42—C41—H41	119.8 (3)
C18—C13—P2	117.5 (3)	C41—C42—C37	120.3 (4)
C18—C13—C14	118.8 (4)	H42—C42—C37	119.8 (2)
H14—C14—C13	119.9 (2)	H42—C42—C41	119.8 (3)
C15—C14—C13	120.2 (4)	O6—C43—O5	123.3 (5)
C15—C14—H14	119.9 (3)	C44—C43—O5	125.8 (4)
H15—C15—C14	119.7 (3)	C44—C43—O6	110.8 (4)
C16—C15—C14	120.6 (4)	H44a—C44—C43	109.5
C16—C15—H15	119.7 (3)	H44b—C44—C43	109.5
H16—C16—C15	120.1 (3)	H44b—C44—H44a	109.5
C17—C16—C15	119.7 (4)	H44c—C44—C43	109.5
C17—C16—H16	120.1 (3)	H44c—C44—H44a	109.5
H17—C17—C16	119.9 (3)	H44c—C44—H44b	109.5
C18—C17—C16	120.1 (4)	H45a—C45—O6	110.5 (3)
C18—C17—H17	119.9 (3)	H45b—C45—O6	110.5 (3)
C17—C18—C13	120.6 (4)	H45b—C45—H45a	108.7
H18—C18—C13	119.7 (2)	C46—C45—O6	106.2 (4)
H18—C18—C17	119.7 (3)	C46—C45—H45a	110.5 (3)
C20—C19—P2	120.8 (3)	C46—C45—H45b	110.5 (3)
C24—C19—P2	119.8 (3)	H46a—C46—C45	109.5
C24—C19—C20	119.4 (4)	H46b—C46—C45	109.5
H20—C20—C19	119.9 (2)	H46b—C46—H46a	109.5
C21—C20—C19	120.1 (4)	H46c—C46—C45	109.5
C21—C20—H20	119.9 (2)	H46c—C46—H46a	109.5
H21—C21—C20	119.8 (2)	H46c—C46—H46b	109.5
			
Pd1—C1—C2—C3	−179.4 (3)	C7—C8—C9—C10	0.1 (5)
Pd1—C1—C6—C5	177.9 (3)	C7—C12—C11—C10	1.4 (5)
P1—C25—C26—C27	−176.8 (4)	C8—C9—C10—C11	−0.9 (5)
P1—C25—C30—C29	177.9 (4)	C9—C10—C11—C12	0.1 (5)
P1—C31—C32—C33	−172.1 (3)	C13—C14—C15—C16	−0.7 (5)
P1—C31—C36—C35	172.4 (3)	C13—C18—C17—C16	−0.6 (5)
P1—C37—C38—C39	−176.9 (3)	C14—C15—C16—C17	2.3 (6)
P1—C37—C42—C41	176.5 (3)	C15—C16—C17—C18	−1.6 (5)
O1—N1—C5—C4	−174.8 (4)	C19—C20—C21—C22	−1.3 (5)
O1—N1—C5—C6	3.6 (5)	C19—C24—C23—C22	−0.4 (5)
N1—C5—C4—C3	177.7 (4)	C20—C21—C22—C23	−1.8 (5)
N1—C5—C6—C1	−176.2 (3)	C21—C22—C23—C24	2.6 (5)
C1—C2—C3—N2	−177.7 (3)	C25—C26—C27—C28	−1.8 (6)
C1—C2—C3—C4	1.1 (4)	C25—C30—C29—C28	−0.1 (6)
C1—C6—C5—C4	2.1 (5)	C26—C27—C28—C29	1.3 (6)
P2—C7—C8—C9	−176.4 (3)	C27—C28—C29—C30	−0.3 (6)
P2—C7—C12—C11	175.5 (3)	C31—C32—C33—C34	−1.1 (5)
P2—C13—C14—C15	173.3 (4)	C31—C36—C35—C34	0.9 (5)
P2—C13—C18—C17	−173.0 (3)	C32—C33—C34—C35	1.6 (5)
P2—C19—C20—C21	−173.5 (3)	C33—C34—C35—C36	−1.4 (5)
P2—C19—C24—C23	174.3 (3)	C37—C38—C39—C40	0.3 (5)
N2—C3—C4—C5	177.8 (4)	C37—C42—C41—C40	0.5 (5)
C2—C3—C4—C5	−0.9 (5)	C38—C39—C40—C41	−0.3 (5)
C3—C4—C5—C6	−0.6 (5)	C39—C40—C41—C42	−0.1 (5)
O5—C43—O6—C45	3.8 (6)		

### (3) Crystal data

**Table:**

[Pd(C_6_H_2_F_3_)I(C_18_H_15_P)_2_]	*F*(000) = 1756.204
*M**_r_* = 888.99	*D*_x_ = 1.694 Mg m^−^^3^
Monoclinic, *I*2/*a*	Mo *K*α radiation, λ = 0.71073 Å
*a* = 11.6327 (5) Å	Cell parameters from 9946 reflections
*b* = 12.8059 (5) Å	θ = 2.4–27.2°
*c* = 23.4327 (9) Å	µ = 1.56 mm^−^^1^
β = 93.218 (2)°	*T* = 100 K
*V* = 3485.2 (2) Å^3^	Irregular, clear orange
*Z* = 4	0.28 × 0.27 × 0.23 mm

### (3) Data collection

**Table:**

Bruker APEXI CCD diffractometer	3735 reflections with *I*≥ 2u(*I*)
φ and ω scans	*R*_int_ = 0.018
Absorption correction: multi-scan (SADABS; Krause *et al.*, 2015)	θ_max_ = 27.2°, θ_min_ = 1.7°
*T*_min_ = 0.705, *T*_max_ = 0.746	*h* = −14→14
22427 measured reflections	*k* = −16→16
3875 independent reflections	*l* = −30→30

### (3) Refinement

**Table:**

Refinement on *F*^2^	39 constraints
Least-squares matrix: full	Primary atom site location: dual
*R*[*F*^2^ > 2σ(*F*^2^)] = 0.021	H-atom parameters constrained
*wR*(*F*^2^) = 0.052	*w* = 1/[σ^2^(*F*_o_^2^) + (0.0197*P*)^2^ + 13.0801*P*] where *P* = (*F*_o_^2^ + 2*F*_c_^2^)/3
*S* = 1.05	(Δ/σ)_max_ = −0.001
3875 reflections	Δρ_max_ = 2.05 e Å^−^^3^
229 parameters	Δρ_min_ = −1.12 e Å^−^^3^
0 restraints	

### (3) Fractional atomic coordinates and isotropic or equivalent isotropic displacement parameters (Å^2^)

**Table:**

	*x*	*y*	*z*	*U*_iso_*/*U*_eq_	Occ. (<1)
Pd1	0.75	0.236973 (16)	0.0	0.01084 (6)	
I1	0.75	0.446148 (14)	0.0	0.01799 (6)	
P1	0.72633 (4)	0.23781 (4)	−0.09914 (2)	0.01123 (10)	
F1	0.9269 (2)	0.0691 (2)	−0.04020 (11)	0.0232 (5)	0.500000
C1	0.75	0.0737 (3)	0.0	0.0271 (5)	
F2	0.9258 (3)	−0.1397 (2)	−0.03982 (14)	0.0356 (7)	0.500000
C2	0.8377 (2)	0.02354 (18)	−0.01838 (9)	0.0252 (5)	0.500000
F3	0.75	−0.24706 (16)	0.0	0.0420 (6)	
C3	0.8418 (2)	−0.08796 (19)	−0.02042 (10)	0.0261 (5)	0.500000
C4	0.75	−0.1412 (3)	0.0	0.0271 (5)	
C5	0.58866 (17)	0.28796 (15)	−0.12888 (8)	0.0139 (4)	
C6	0.51028 (18)	0.33479 (16)	−0.09401 (9)	0.0172 (4)	
H6	0.53051 (18)	0.34601 (16)	−0.05465 (9)	0.0207 (5)*	
C7	0.40197 (19)	0.36523 (18)	−0.11691 (10)	0.0221 (4)	
H7	0.34844 (19)	0.39684 (18)	−0.09309 (10)	0.0265 (5)*	
C8	0.37264 (19)	0.34931 (17)	−0.17438 (11)	0.0219 (5)	
H8	0.29915 (19)	0.37059 (17)	−0.18991 (11)	0.0263 (5)*	
C9	0.45004 (18)	0.30242 (17)	−0.20941 (9)	0.0194 (4)	
H9	0.42961 (18)	0.29169 (17)	−0.24880 (9)	0.0233 (5)*	
C10	0.55753 (18)	0.27125 (16)	−0.18665 (9)	0.0164 (4)	
H10	0.61015 (18)	0.23839 (16)	−0.21050 (9)	0.0197 (5)*	
C11	0.73091 (17)	0.11054 (16)	−0.13442 (8)	0.0135 (4)	
C12	0.82437 (18)	0.07795 (17)	−0.16450 (9)	0.0181 (4)	
H12	0.88437 (18)	0.12567 (17)	−0.17173 (9)	0.0217 (5)*	
C13	0.82994 (19)	−0.02488 (19)	−0.18406 (9)	0.0229 (5)	
H13	0.89408 (19)	−0.04698 (19)	−0.20434 (9)	0.0275 (5)*	
C14	0.7428 (2)	−0.09460 (18)	−0.17409 (10)	0.0243 (5)	
H14	0.7476 (2)	−0.16478 (18)	−0.18687 (10)	0.0291 (6)*	
C15	0.64770 (19)	−0.06188 (17)	−0.14527 (10)	0.0212 (4)	
H15	0.58697 (19)	−0.10942 (17)	−0.13905 (10)	0.0255 (5)*	
C16	0.64151 (18)	0.04002 (16)	−0.12563 (9)	0.0169 (4)	
H16	0.57635 (18)	0.06211 (16)	−0.10612 (9)	0.0202 (5)*	
C17	0.84344 (17)	0.31007 (16)	−0.12955 (8)	0.0141 (4)	
C18	0.83228 (18)	0.36272 (16)	−0.18177 (9)	0.0167 (4)	
H18	0.76010 (18)	0.36412 (16)	−0.20284 (9)	0.0201 (5)*	
C19	0.9272 (2)	0.41326 (17)	−0.20295 (9)	0.0208 (4)	
H19	0.9197 (2)	0.44869 (17)	−0.23858 (9)	0.0249 (5)*	
C20	1.0326 (2)	0.41195 (18)	−0.17216 (10)	0.0230 (5)	
H20	1.0967 (2)	0.44704 (18)	−0.18663 (10)	0.0276 (5)*	
C21	1.04467 (19)	0.35962 (19)	−0.12031 (10)	0.0233 (5)	
H21	1.11705 (19)	0.35847 (19)	−0.09942 (10)	0.0279 (5)*	
C22	0.95025 (18)	0.30879 (18)	−0.09903 (9)	0.0186 (4)	
H22	0.95847 (18)	0.27299 (18)	−0.06353 (9)	0.0224 (5)*	
C2A	0.8377 (2)	0.02354 (18)	−0.01838 (9)	0.0252 (5)	0.500000
H2A	0.9012 (2)	0.06228 (18)	−0.03091 (9)	0.0302 (6)*	0.500000
C3A	0.8418 (2)	−0.08796 (19)	−0.02042 (10)	0.0261 (5)	0.500000
H3A	0.9052 (2)	−0.12371 (19)	−0.03523 (10)	0.0314 (6)*	0.500000

### (3) Atomic displacement parameters (Å^2^)

**Table:**

	*U*^11^	*U*^22^	*U*^33^	*U*^12^	*U*^13^	*U*^23^
Pd1	0.01480 (10)	0.01060 (10)	0.00714 (10)	−0.000000	0.00076 (7)	0.000000
I1	0.02728 (11)	0.01232 (9)	0.01457 (9)	−0.000000	0.00294 (7)	0.000000
P1	0.0126 (2)	0.0130 (2)	0.0082 (2)	0.00102 (18)	0.00100 (17)	−0.00025 (17)
F1	0.0175 (12)	0.0258 (14)	0.0265 (14)	0.0013 (10)	0.0030 (10)	0.0015 (11)
C1	0.0407 (14)	0.0293 (12)	0.0107 (10)	−0.000000	−0.0045 (9)	0.000000
F2	0.0361 (16)	0.0242 (15)	0.0472 (19)	0.0111 (13)	0.0093 (14)	−0.0015 (13)
C2	0.0326 (12)	0.0223 (11)	0.0185 (10)	−0.0098 (9)	−0.0179 (9)	0.0149 (9)
F3	0.0635 (16)	0.0150 (10)	0.0486 (14)	−0.000000	0.0128 (12)	0.000000
C3	0.0353 (13)	0.0199 (11)	0.0232 (11)	0.0072 (10)	0.0013 (10)	−0.0018 (9)
C4	0.0407 (14)	0.0293 (12)	0.0107 (10)	−0.000000	−0.0045 (9)	0.000000
C5	0.0143 (9)	0.0121 (9)	0.0150 (9)	0.0004 (7)	−0.0004 (7)	0.0022 (7)
C6	0.0176 (10)	0.0169 (10)	0.0175 (10)	−0.0013 (8)	0.0035 (8)	−0.0003 (8)
C7	0.0159 (10)	0.0193 (10)	0.0316 (12)	0.0024 (8)	0.0051 (9)	−0.0025 (9)
C8	0.0151 (10)	0.0155 (10)	0.0344 (12)	0.0010 (8)	−0.0043 (9)	0.0027 (9)
C9	0.0201 (10)	0.0176 (10)	0.0198 (10)	−0.0005 (8)	−0.0056 (8)	0.0020 (8)
C10	0.0168 (10)	0.0165 (10)	0.0158 (10)	0.0019 (8)	0.0005 (8)	0.0002 (8)
C11	0.0157 (9)	0.0146 (9)	0.0097 (8)	0.0037 (7)	−0.0031 (7)	−0.0016 (7)
C12	0.0171 (10)	0.0228 (11)	0.0143 (9)	0.0022 (8)	0.0004 (8)	−0.0025 (8)
C13	0.0201 (10)	0.0286 (12)	0.0198 (10)	0.0095 (9)	−0.0010 (8)	−0.0089 (9)
C14	0.0263 (11)	0.0199 (11)	0.0253 (11)	0.0082 (9)	−0.0101 (9)	−0.0099 (9)
C15	0.0203 (10)	0.0174 (10)	0.0251 (11)	0.0003 (8)	−0.0071 (8)	−0.0027 (8)
C16	0.0158 (9)	0.0187 (10)	0.0159 (9)	0.0018 (8)	−0.0011 (7)	−0.0017 (8)
C17	0.0161 (9)	0.0147 (9)	0.0117 (9)	−0.0003 (7)	0.0034 (7)	−0.0021 (7)
C18	0.0206 (10)	0.0160 (9)	0.0138 (9)	0.0001 (8)	0.0022 (8)	−0.0014 (8)
C19	0.0291 (11)	0.0163 (10)	0.0177 (10)	−0.0013 (9)	0.0086 (8)	0.0003 (8)
C20	0.0222 (11)	0.0208 (11)	0.0271 (11)	−0.0047 (9)	0.0122 (9)	−0.0040 (9)
C21	0.0160 (10)	0.0271 (12)	0.0270 (11)	−0.0021 (9)	0.0033 (9)	−0.0050 (9)
C22	0.0174 (10)	0.0228 (11)	0.0158 (10)	−0.0003 (8)	0.0012 (8)	0.0004 (8)
C2A	0.0326 (12)	0.0223 (11)	0.0185 (10)	−0.0098 (9)	−0.0179 (9)	0.0149 (9)
C3A	0.0353 (13)	0.0199 (11)	0.0232 (11)	0.0072 (10)	0.0013 (10)	−0.0018 (9)

### (3) Geometric parameters (Å, º)

**Table:**

Pd1—I1	2.6787 (3)	C9—C10	1.390 (3)
Pd1—P1^i^	2.3239 (5)	C10—H10	0.9500
Pd1—P1	2.3239 (5)	C11—C12	1.392 (3)
Pd1—C1	2.091 (4)	C11—C16	1.401 (3)
P1—C5	1.827 (2)	C12—H12	0.9500
P1—C11	1.830 (2)	C12—C13	1.397 (3)
P1—C17	1.825 (2)	C13—H13	0.9500
F1—C2	1.319 (3)	C13—C14	1.381 (4)
C1—C2^i^	1.299 (3)	C14—H14	0.9500
C1—C2	1.299 (3)	C14—C15	1.392 (3)
C1—C2A	1.299 (3)	C15—H15	0.9500
C1—C2A^i^	1.299 (3)	C15—C16	1.387 (3)
F2—C3	1.284 (4)	C16—H16	0.9500
C2—C3	1.430 (3)	C17—C18	1.397 (3)
F3—C4	1.356 (4)	C17—C22	1.399 (3)
C3—C4^i^	1.375 (3)	C18—H18	0.9500
C4—C3A^i^	1.375 (3)	C18—C19	1.395 (3)
C4—C3A	1.375 (3)	C19—H19	0.9500
C5—C6	1.394 (3)	C19—C20	1.387 (3)
C5—C10	1.398 (3)	C20—H20	0.9500
C6—H6	0.9500	C20—C21	1.388 (3)
C6—C7	1.397 (3)	C21—H21	0.9500
C7—H7	0.9500	C21—C22	1.393 (3)
C7—C8	1.386 (3)	C22—H22	0.9500
C8—H8	0.9500	C2A—H2A	0.9500
C8—C9	1.389 (3)	C2A—C3A	1.430 (3)
C9—H9	0.9500	C3A—H3A	0.9500
			
P1—Pd1—I1	89.737 (13)	C9—C8—H8	119.81 (13)
P1^i^—Pd1—I1	89.737 (13)	H9—C9—C8	120.12 (13)
P1^i^—Pd1—P1	179.47 (3)	C10—C9—C8	119.8 (2)
C1—Pd1—I1	180.0	C10—C9—H9	120.12 (13)
C1—Pd1—P1	90.263 (13)	C9—C10—C5	120.41 (19)
C1—Pd1—P1^i^	90.263 (13)	H10—C10—C5	119.80 (12)
C5—P1—Pd1	115.79 (7)	H10—C10—C9	119.80 (13)
C11—P1—Pd1	116.28 (6)	C12—C11—P1	122.62 (16)
C11—P1—C5	100.83 (9)	C16—C11—P1	117.84 (15)
C17—P1—Pd1	110.10 (7)	C16—C11—C12	119.23 (19)
C17—P1—C5	109.29 (9)	H12—C12—C11	119.99 (12)
C17—P1—C11	103.53 (9)	C13—C12—C11	120.0 (2)
C2—C1—Pd1	119.64 (17)	C13—C12—H12	119.99 (13)
C2^i^—C1—Pd1	119.64 (17)	H13—C13—C12	119.80 (13)
C2^i^—C1—C2	120.7 (3)	C14—C13—C12	120.4 (2)
C2A^i^—C1—Pd1	119.64 (17)	C14—C13—H13	119.80 (13)
C2A—C1—Pd1	119.64 (17)	H14—C14—C13	120.05 (13)
C2A—C1—C2^i^	120.7 (3)	C15—C14—C13	119.9 (2)
C2A^i^—C1—C2^i^	0.0	C15—C14—H14	120.05 (13)
C2A^i^—C1—C2	120.7 (3)	H15—C15—C14	119.95 (13)
C2A—C1—C2	0.0	C16—C15—C14	120.1 (2)
C2A^i^—C1—C2A	120.7 (3)	C16—C15—H15	119.95 (13)
C1—C2—F1	124.0 (3)	C15—C16—C11	120.3 (2)
C3—C2—F1	113.6 (3)	H16—C16—C11	119.84 (12)
C3—C2—C1	122.3 (2)	H16—C16—C15	119.84 (13)
C2—C3—F2	123.7 (3)	C18—C17—P1	123.52 (16)
C4^i^—C3—F2	119.3 (3)	C22—C17—P1	117.11 (15)
C4^i^—C3—C2	117.0 (2)	C22—C17—C18	119.33 (19)
C3^i^—C4—F3	119.69 (16)	H18—C18—C17	120.03 (12)
C3—C4—F3	119.69 (16)	C19—C18—C17	119.9 (2)
C3—C4—C3^i^	120.6 (3)	C19—C18—H18	120.03 (13)
C3A—C4—F3	119.69 (16)	H19—C19—C18	119.89 (13)
C3A^i^—C4—F3	119.69 (16)	C20—C19—C18	120.2 (2)
C3A^i^—C4—C3	120.6 (3)	C20—C19—H19	119.89 (13)
C3A^i^—C4—C3^i^	0.0	H20—C20—C19	119.85 (13)
C3A—C4—C3^i^	120.6 (3)	C21—C20—C19	120.3 (2)
C3A—C4—C3	0.0	C21—C20—H20	119.85 (13)
C3A—C4—C3A^i^	120.6 (3)	H21—C21—C20	120.14 (13)
C6—C5—P1	121.06 (15)	C22—C21—C20	119.7 (2)
C10—C5—P1	119.31 (15)	C22—C21—H21	120.14 (13)
C10—C5—C6	119.41 (19)	C21—C22—C17	120.5 (2)
H6—C6—C5	119.98 (12)	H22—C22—C17	119.76 (12)
C7—C6—C5	120.0 (2)	H22—C22—C21	119.76 (13)
C7—C6—H6	119.98 (13)	H2A—C2A—C1	118.87 (17)
H7—C7—C6	120.01 (13)	C3A—C2A—C1	122.3 (2)
C8—C7—C6	120.0 (2)	C3A—C2A—H2A	118.87 (15)
C8—C7—H7	120.01 (13)	C2A—C3A—C4^i^	117.0 (2)
H8—C8—C7	119.81 (13)	H3A—C3A—C4^i^	121.48 (16)
C9—C8—C7	120.4 (2)	H3A—C3A—C2A	121.48 (15)
			
Pd1—C1—C2—F1	−3.43 (14)	F2—C3—C4^i^—F3^i^	0.8 (3)
Pd1—C1—C2^i^—F1^i^	−3.43 (14)	F2—C3—C4^i^—C3^i^	−179.22 (18)
Pd1—C1—C2—C3	−178.84 (15)	F2—C3—C4^i^—C3A^i^	−179.22 (18)
Pd1—C1—C2^i^—C3^i^	−178.84 (15)	C2—C3—C4^i^—F3^i^	−178.96 (14)
Pd1—C1—C2A^i^—C3A^i^	−178.84 (15)	C2—C3—C4^i^—C3^i^	1.0 (3)
Pd1—C1—C2A—C3A	−178.84 (15)	C2—C3—C4^i^—C3A^i^	1.0 (3)
P1—C5—C6—C7	−175.03 (17)	F3—C4—C3A^i^—C2A^i^	−178.96 (15)
P1—C5—C10—C9	175.67 (16)	F3—C4—C3A—C2A	−178.96 (15)
P1—C11—C12—C13	171.50 (17)	C3^i^—C4—C3A—C2A	1.04 (15)
P1—C11—C16—C15	−171.90 (15)	C5—C6—C7—C8	−0.3 (2)
P1—C17—C18—C19	−177.85 (17)	C5—C10—C9—C8	−0.8 (2)
P1—C17—C22—C21	178.16 (16)	C6—C7—C8—C9	0.5 (3)
F1—C2—C1—C2^i^	176.57 (13)	C7—C8—C9—C10	0.0 (3)
F1—C2—C1—C2A^i^	176.57 (14)	C11—C12—C13—C14	0.4 (2)
F1—C2—C3—F2	2.2 (3)	C11—C16—C15—C14	−0.3 (2)
F1—C2—C3—C4^i^	−178.11 (19)	C12—C13—C14—C15	1.1 (3)
C1—C2—C3—F2	178.0 (3)	C13—C14—C15—C16	−1.2 (3)
C1^i^—C2—C3—F2	178.0 (3)	C17—C18—C19—C20	−0.4 (2)
C1—C2—C3—C4^i^	−2.3 (2)	C17—C22—C21—C20	0.0 (3)
C1—C2^i^—C3^i^—C4	−2.3 (2)	C18—C19—C20—C21	0.6 (3)
C1—C2A—C3A—C4^i^	−2.3 (2)	C19—C20—C21—C22	−0.4 (3)
C1—C2A^i^—C3A^i^—C4	−2.3 (2)		

Symmetry code: (i) −*x*+3/2, *y*, −*z*.

### (4) Crystal data

**Table:**

[Pd(C_6_H_2_F_3_)I(C_18_H_15_P)_2_]·2CH_2_Cl_2_	*Z* = 2
*M**_r_* = 1310.64	*F*(000) = 1253.792
Triclinic, *P*1	*D*_x_ = 1.888 Mg m^−^^3^
*a* = 11.5608 (5) Å	Mo *K*α radiation, λ = 0.71073 Å
*b* = 11.6437 (5) Å	Cell parameters from 9954 reflections
*c* = 18.1311 (8) Å	θ = 2.4–27.2°
α = 76.426 (1)°	µ = 2.76 mm^−^^1^
β = 77.722 (1)°	*T* = 100 K
γ = 81.344 (1)°	Block, clear orange
*V* = 2305.16 (17) Å^3^	0.23 × 0.11 × 0.10 mm

### (4) Data collection

**Table:**

Bruker APEXI CCD diffractometer	8450 reflections with *I*≥ 2u(*I*)
φ and ω scans	*R*_int_ = 0.030
Absorption correction: multi-scan (SADABS; Krause *et al.*, 2015)	θ_max_ = 27.2°, θ_min_ = 1.2°
*T*_min_ = 0.674, *T*_max_ = 0.746	*h* = −14→14
28953 measured reflections	*k* = −14→14
10132 independent reflections	*l* = −23→23

### (4) Refinement

**Table:**

Refinement on *F*^2^	70 constraints
Least-squares matrix: full	Primary atom site location: iterative
*R*[*F*^2^ > 2σ(*F*^2^)] = 0.030	H-atom parameters constrained
*wR*(*F*^2^) = 0.062	*w* = 1/[σ^2^(*F*_o_^2^) + (0.019*P*)^2^ + 4.2389*P*] where *P* = (*F*_o_^2^ + 2*F*_c_^2^)/3
*S* = 1.06	(Δ/σ)_max_ = −0.0002
10132 reflections	Δρ_max_ = 1.06 e Å^−^^3^
542 parameters	Δρ_min_ = −1.03 e Å^−^^3^
2 restraints	

### (4) Fractional atomic coordinates and isotropic or equivalent isotropic displacement parameters (Å^2^)

**Table:**

	*x*	*y*	*z*	*U*_iso_*/*U*_eq_	Occ. (<1)
I1	0.23962 (2)	−0.04364 (2)	0.796612 (13)	0.01617 (6)	
Pd1	0.39999 (2)	0.11224 (2)	0.758753 (14)	0.00837 (6)	
Cl1	0.01465 (12)	0.81829 (14)	0.58804 (10)	0.0617 (4)	
P1	0.25283 (8)	0.26815 (8)	0.77555 (5)	0.01070 (17)	
F1	0.50264 (18)	0.22392 (18)	0.58875 (11)	0.0185 (4)	
C1	0.5197 (3)	0.2311 (3)	0.71437 (19)	0.0106 (7)	
I2	0.73642 (2)	0.43117 (2)	0.798995 (15)	0.02142 (6)	
Cl2	0.20648 (11)	0.97093 (10)	0.54543 (7)	0.0409 (3)	
P2	0.55621 (8)	−0.03829 (7)	0.75689 (5)	0.00942 (17)	
F2	0.76543 (18)	0.47459 (18)	0.61696 (12)	0.0205 (5)	
C2	0.5527 (3)	0.2700 (3)	0.63565 (19)	0.0123 (7)	
I3	0.68119 (2)	0.40425 (2)	0.481715 (14)	0.02607 (7)	
F3	0.54666 (18)	0.24532 (18)	0.83631 (11)	0.0170 (4)	
C3	0.6339 (3)	0.3512 (3)	0.60091 (19)	0.0138 (7)	
Cl4A	−0.0440 (4)	0.6948 (4)	0.7824 (4)	0.0638 (14)	0.631 (13)
C4	0.6853 (3)	0.3955 (3)	0.6492 (2)	0.0139 (7)	
C5	0.6578 (3)	0.3611 (3)	0.7285 (2)	0.0130 (7)	
C6	0.5755 (3)	0.2792 (3)	0.75843 (18)	0.0110 (7)	
C7	0.7032 (3)	0.0088 (3)	0.74620 (19)	0.0121 (7)	
C8	0.7531 (3)	0.0083 (3)	0.8098 (2)	0.0194 (8)	
H8	0.7123 (3)	−0.0217 (3)	0.8604 (2)	0.0233 (10)*	
C9	0.8621 (4)	0.0516 (4)	0.7993 (2)	0.0300 (10)	
H9	0.8964 (4)	0.0503 (4)	0.8428 (2)	0.0361 (12)*	
C10	0.9203 (3)	0.0962 (4)	0.7265 (2)	0.0283 (9)	
H10	0.9946 (3)	0.1263 (4)	0.7199 (2)	0.0340 (11)*	
C11	0.8735 (3)	0.0983 (3)	0.6630 (2)	0.0227 (8)	
H11	0.9149 (3)	0.1297 (3)	0.6128 (2)	0.0272 (10)*	
C12	0.7645 (3)	0.0541 (3)	0.6724 (2)	0.0162 (7)	
H12	0.7318 (3)	0.0549 (3)	0.6284 (2)	0.0194 (9)*	
C13	0.5396 (3)	−0.1438 (3)	0.84997 (19)	0.0137 (7)	
C14	0.4838 (3)	−0.0990 (3)	0.9152 (2)	0.0213 (8)	
H14	0.4542 (3)	−0.0174 (3)	0.9098 (2)	0.0255 (10)*	
C15	0.4714 (4)	−0.1726 (4)	0.9874 (2)	0.0284 (9)	
H15	0.4339 (4)	−0.1412 (4)	1.0314 (2)	0.0341 (11)*	
C16	0.5131 (4)	−0.2918 (4)	0.9961 (2)	0.0310 (10)	
H16	0.5049 (4)	−0.3424 (4)	1.0459 (2)	0.0372 (12)*	
C17	0.5667 (5)	−0.3361 (4)	0.9316 (2)	0.0376 (12)	
H17	0.5948 (5)	−0.4181 (4)	0.9371 (2)	0.0451 (14)*	
C18	0.5802 (4)	−0.2629 (3)	0.8586 (2)	0.0247 (9)	
H18	0.6175 (4)	−0.2948 (3)	0.8147 (2)	0.0296 (11)*	
C19	0.5783 (3)	−0.1195 (3)	0.68008 (18)	0.0122 (7)	
C20	0.6813 (3)	−0.1969 (3)	0.66570 (19)	0.0142 (7)	
H20	0.7392 (3)	−0.2099 (3)	0.69779 (19)	0.0170 (9)*	
C21	0.6996 (3)	−0.2548 (3)	0.6051 (2)	0.0173 (8)	
H21	0.7687 (3)	−0.3090 (3)	0.5966 (2)	0.0207 (9)*	
C22	0.6172 (3)	−0.2338 (3)	0.5567 (2)	0.0184 (8)	
H22	0.6301 (3)	−0.2737 (3)	0.5152 (2)	0.0221 (9)*	
C23	0.5160 (3)	−0.1550 (3)	0.5687 (2)	0.0168 (8)	
H23	0.4604 (3)	−0.1399 (3)	0.5350 (2)	0.0202 (9)*	
C24	0.4964 (3)	−0.0981 (3)	0.63045 (19)	0.0137 (7)	
H24	0.4269 (3)	−0.0443 (3)	0.63891 (19)	0.0165 (8)*	
C25	0.1474 (3)	0.2264 (3)	0.86487 (19)	0.0151 (7)	
C26	0.0249 (3)	0.2414 (3)	0.8695 (2)	0.0221 (8)	
H26	−0.0084 (3)	0.2767 (3)	0.8248 (2)	0.0265 (10)*	
C27	−0.0490 (4)	0.2045 (4)	0.9401 (2)	0.0325 (10)	
H27	−0.1329 (4)	0.2148 (4)	0.9432 (2)	0.0390 (12)*	
C28	−0.0023 (4)	0.1533 (4)	1.0051 (2)	0.0390 (11)	
H28	−0.0534 (4)	0.1277 (4)	1.0529 (2)	0.0467 (14)*	
C29	0.1199 (4)	0.1393 (5)	1.0006 (2)	0.0401 (12)	
H29	0.1527 (4)	0.1048 (5)	1.0456 (2)	0.0481 (14)*	
C30	0.1942 (4)	0.1749 (4)	0.9313 (2)	0.0283 (9)	
H30	0.2780 (4)	0.1644 (4)	0.9287 (2)	0.0340 (11)*	
C31	0.1702 (3)	0.3145 (3)	0.69683 (19)	0.0131 (7)	
C32	0.0913 (3)	0.4180 (3)	0.6909 (2)	0.0171 (8)	
H32	0.0820 (3)	0.4666 (3)	0.7276 (2)	0.0205 (9)*	
C33	0.0262 (3)	0.4504 (3)	0.6318 (2)	0.0197 (8)	
H33	−0.0269 (3)	0.5213 (3)	0.6278 (2)	0.0236 (10)*	
C34	0.0389 (3)	0.3787 (3)	0.5786 (2)	0.0212 (8)	
H34	−0.0076 (3)	0.3992 (3)	0.5392 (2)	0.0254 (10)*	
C35	0.1194 (3)	0.2776 (3)	0.5830 (2)	0.0168 (8)	
H35	0.1296 (3)	0.2301 (3)	0.5455 (2)	0.0202 (9)*	
C36	0.1853 (3)	0.2451 (3)	0.64186 (19)	0.0141 (7)	
H36	0.2405 (3)	0.1757 (3)	0.64457 (19)	0.0169 (9)*	
C37	0.2962 (3)	0.4087 (3)	0.7839 (2)	0.0151 (7)	
C38	0.2716 (4)	0.4474 (4)	0.8533 (3)	0.0317 (10)	
H38	0.2304 (4)	0.4007 (4)	0.8987 (3)	0.0381 (12)*	
C39	0.3080 (5)	0.5555 (4)	0.8552 (3)	0.0492 (14)	
H39	0.2930 (5)	0.5815 (4)	0.9025 (3)	0.0591 (16)*	
C40	0.3655 (4)	0.6247 (4)	0.7891 (3)	0.0398 (12)	
H40	0.3880 (4)	0.6991 (4)	0.7908 (3)	0.0477 (14)*	
C41	0.3898 (3)	0.5870 (3)	0.7221 (3)	0.0256 (9)	
H41	0.4308 (3)	0.6346 (3)	0.6770 (3)	0.0307 (11)*	
C42	0.3557 (3)	0.4791 (3)	0.7181 (2)	0.0205 (8)	
H42	0.3731 (3)	0.4537 (3)	0.6704 (2)	0.0246 (10)*	
C43	0.1318 (4)	0.8661 (4)	0.6189 (3)	0.0387 (11)	
H43a	0.0988 (4)	0.9016 (4)	0.6645 (3)	0.0465 (13)*	
H43b	0.1890 (4)	0.7967 (4)	0.6343 (3)	0.0465 (13)*	
C44A	−0.0609 (15)	0.6592 (12)	0.8844 (7)	0.102 (6)	0.631 (13)
H44a	0.0120 (15)	0.6736 (12)	0.8997 (7)	0.122 (7)*	0.631 (13)
H44b	−0.1285 (15)	0.7109 (12)	0.9070 (7)	0.122 (7)*	0.631 (13)
Cl3A	−0.0864 (6)	0.5108 (4)	0.9195 (3)	0.0742 (17)	0.631 (13)
Cl3B	−0.1518 (10)	0.5447 (9)	0.9410 (3)	0.075 (3)	0.369 (13)
C44B	−0.0159 (17)	0.5878 (18)	0.8910 (11)	0.059 (5)	0.369 (13)
H44c	0.0218 (17)	0.6221 (18)	0.9239 (11)	0.071 (6)*	0.369 (13)
H44d	0.0370 (17)	0.5178 (18)	0.8774 (11)	0.071 (6)*	0.369 (13)
Cl4B	−0.0338 (13)	0.6933 (13)	0.8068 (9)	0.126 (5)	0.369 (13)

### (4) Atomic displacement parameters (Å^2^)

**Table:**

	*U*^11^	*U*^22^	*U*^33^	*U*^12^	*U*^13^	*U*^23^
I1	0.01382 (11)	0.01547 (12)	0.01843 (12)	−0.00637 (9)	0.00093 (9)	−0.00247 (9)
Pd1	0.00817 (12)	0.00891 (12)	0.00759 (12)	−0.00203 (9)	−0.00059 (9)	−0.00098 (9)
Cl1	0.0393 (7)	0.0703 (10)	0.0906 (11)	−0.0080 (7)	−0.0169 (7)	−0.0409 (9)
P1	0.0100 (4)	0.0108 (4)	0.0106 (4)	−0.0008 (3)	−0.0007 (3)	−0.0020 (3)
F1	0.0241 (11)	0.0255 (11)	0.0092 (10)	−0.0116 (9)	−0.0039 (8)	−0.0038 (9)
C1	0.0092 (16)	0.0102 (16)	0.0113 (16)	0.0003 (13)	−0.0019 (13)	−0.0008 (13)
I2	0.01897 (13)	0.02511 (13)	0.02632 (14)	−0.00558 (10)	−0.00724 (10)	−0.01266 (11)
Cl2	0.0398 (7)	0.0379 (6)	0.0457 (7)	0.0025 (5)	−0.0111 (5)	−0.0115 (5)
P2	0.0102 (4)	0.0100 (4)	0.0081 (4)	−0.0017 (3)	−0.0017 (3)	−0.0017 (3)
F2	0.0162 (11)	0.0169 (11)	0.0272 (12)	−0.0108 (9)	0.0011 (9)	−0.0011 (9)
C2	0.0125 (17)	0.0154 (17)	0.0112 (17)	−0.0021 (14)	−0.0042 (13)	−0.0046 (14)
I3	0.03102 (15)	0.02993 (15)	0.01275 (12)	−0.00971 (12)	0.00400 (10)	0.00125 (10)
F3	0.0216 (11)	0.0233 (11)	0.0072 (10)	−0.0054 (9)	−0.0037 (8)	−0.0024 (8)
C3	0.0151 (18)	0.0148 (17)	0.0087 (16)	−0.0029 (14)	0.0008 (13)	0.0014 (14)
Cl4A	0.0508 (19)	0.0495 (19)	0.100 (4)	−0.0058 (13)	−0.0155 (19)	−0.032 (2)
C4	0.0099 (17)	0.0106 (17)	0.0187 (18)	−0.0035 (13)	0.0005 (14)	0.0006 (14)
C5	0.0125 (17)	0.0112 (16)	0.0173 (18)	−0.0009 (13)	−0.0031 (14)	−0.0070 (14)
C6	0.0127 (17)	0.0114 (16)	0.0078 (16)	0.0016 (13)	−0.0022 (13)	−0.0015 (13)
C7	0.0097 (16)	0.0105 (16)	0.0163 (17)	0.0003 (13)	−0.0042 (13)	−0.0025 (14)
C8	0.022 (2)	0.0188 (19)	0.0174 (19)	−0.0092 (16)	−0.0094 (15)	0.0056 (15)
C9	0.028 (2)	0.037 (2)	0.028 (2)	−0.0146 (19)	−0.0171 (18)	0.0039 (19)
C10	0.014 (2)	0.032 (2)	0.039 (2)	−0.0072 (17)	−0.0096 (18)	−0.0002 (19)
C11	0.0165 (19)	0.021 (2)	0.026 (2)	−0.0060 (16)	0.0039 (16)	−0.0015 (17)
C12	0.0179 (19)	0.0169 (18)	0.0148 (18)	−0.0027 (15)	−0.0041 (14)	−0.0039 (14)
C13	0.0157 (18)	0.0155 (17)	0.0118 (17)	−0.0038 (14)	−0.0045 (14)	−0.0035 (14)
C14	0.030 (2)	0.0192 (19)	0.0122 (18)	0.0031 (17)	−0.0036 (16)	−0.0028 (15)
C15	0.045 (3)	0.028 (2)	0.0106 (19)	0.0050 (19)	−0.0049 (17)	−0.0056 (16)
C16	0.059 (3)	0.021 (2)	0.0093 (19)	−0.008 (2)	−0.0032 (19)	0.0037 (16)
C17	0.078 (4)	0.0120 (19)	0.018 (2)	0.000 (2)	−0.005 (2)	−0.0003 (16)
C18	0.044 (3)	0.0155 (19)	0.0116 (18)	0.0013 (18)	−0.0008 (17)	−0.0042 (15)
C19	0.0153 (17)	0.0122 (17)	0.0076 (16)	−0.0065 (14)	0.0022 (13)	0.0001 (13)
C20	0.0163 (18)	0.0121 (17)	0.0131 (17)	−0.0001 (14)	−0.0027 (14)	−0.0016 (14)
C21	0.0181 (19)	0.0129 (17)	0.0182 (19)	0.0005 (15)	0.0002 (15)	−0.0030 (15)
C22	0.024 (2)	0.0210 (19)	0.0126 (18)	−0.0084 (16)	−0.0002 (15)	−0.0070 (15)
C23	0.0173 (19)	0.0218 (19)	0.0137 (18)	−0.0048 (15)	−0.0039 (14)	−0.0056 (15)
C24	0.0130 (17)	0.0151 (17)	0.0130 (17)	−0.0033 (14)	−0.0015 (13)	−0.0026 (14)
C25	0.0173 (18)	0.0138 (17)	0.0117 (17)	−0.0020 (14)	0.0027 (14)	−0.0021 (14)
C26	0.018 (2)	0.029 (2)	0.0168 (19)	−0.0036 (16)	0.0003 (15)	−0.0032 (16)
C27	0.020 (2)	0.046 (3)	0.028 (2)	−0.010 (2)	0.0079 (17)	−0.007 (2)
C28	0.045 (3)	0.048 (3)	0.016 (2)	−0.014 (2)	0.0110 (19)	−0.001 (2)
C29	0.039 (3)	0.059 (3)	0.014 (2)	−0.001 (2)	−0.0023 (19)	0.005 (2)
C30	0.024 (2)	0.038 (2)	0.015 (2)	0.0021 (19)	0.0004 (16)	0.0026 (17)
C31	0.0090 (16)	0.0174 (18)	0.0106 (17)	−0.0035 (14)	−0.0019 (13)	0.0030 (14)
C32	0.0145 (18)	0.0172 (18)	0.0181 (19)	−0.0027 (15)	0.0000 (14)	−0.0027 (15)
C33	0.0122 (18)	0.0182 (19)	0.024 (2)	0.0009 (15)	−0.0034 (15)	0.0023 (16)
C34	0.0178 (19)	0.028 (2)	0.0144 (18)	−0.0074 (16)	−0.0062 (15)	0.0085 (16)
C35	0.0138 (18)	0.024 (2)	0.0122 (17)	−0.0068 (15)	0.0002 (14)	−0.0033 (15)
C36	0.0133 (17)	0.0165 (18)	0.0097 (16)	−0.0039 (14)	0.0009 (13)	0.0015 (14)
C37	0.0135 (17)	0.0095 (16)	0.0232 (19)	0.0020 (14)	−0.0070 (15)	−0.0038 (14)
C38	0.045 (3)	0.025 (2)	0.028 (2)	−0.008 (2)	−0.003 (2)	−0.0118 (18)
C39	0.073 (4)	0.038 (3)	0.048 (3)	−0.009 (3)	−0.017 (3)	−0.025 (3)
C40	0.046 (3)	0.017 (2)	0.063 (3)	−0.009 (2)	−0.018 (3)	−0.012 (2)
C41	0.0158 (19)	0.0082 (17)	0.048 (3)	−0.0021 (15)	−0.0053 (18)	0.0041 (17)
C42	0.0180 (19)	0.0129 (18)	0.027 (2)	0.0014 (15)	−0.0041 (16)	0.0003 (16)
C43	0.035 (3)	0.040 (3)	0.046 (3)	−0.001 (2)	−0.011 (2)	−0.016 (2)
C44A	0.147 (15)	0.091 (11)	0.107 (12)	0.007 (10)	−0.064 (10)	−0.071 (10)
Cl3A	0.080 (4)	0.091 (2)	0.065 (3)	0.006 (2)	−0.039 (3)	−0.0290 (18)
Cl3B	0.067 (5)	0.104 (5)	0.064 (3)	−0.013 (4)	−0.004 (3)	−0.042 (3)
C44B	0.062 (12)	0.044 (11)	0.087 (13)	−0.003 (9)	−0.023 (10)	−0.041 (10)
Cl4B	0.171 (10)	0.130 (8)	0.088 (8)	−0.062 (7)	−0.020 (6)	−0.018 (5)

### (4) Geometric parameters (Å, º)

**Table:**

I1—Pd1	2.6626 (3)	C21—H21	0.9500
Pd1—P1	2.3221 (9)	C21—C22	1.387 (5)
Pd1—C1	2.014 (3)	C22—H22	0.9500
Pd1—P2	2.3203 (9)	C22—C23	1.385 (5)
Cl1—C43	1.775 (5)	C23—H23	0.9500
P1—C25	1.820 (3)	C23—C24	1.393 (5)
P1—C31	1.818 (3)	C24—H24	0.9500
P1—C37	1.830 (3)	C25—C26	1.387 (5)
F1—C2	1.365 (4)	C25—C30	1.395 (5)
C1—C2	1.380 (5)	C26—H26	0.9500
C1—C6	1.381 (4)	C26—C27	1.393 (5)
I2—C5	2.080 (3)	C27—H27	0.9500
Cl2—C43	1.753 (5)	C27—C28	1.372 (6)
P2—C7	1.821 (3)	C28—H28	0.9500
P2—C13	1.831 (3)	C28—C29	1.385 (6)
P2—C19	1.815 (3)	C29—H29	0.9500
F2—C4	1.349 (4)	C29—C30	1.375 (6)
C2—C3	1.382 (5)	C30—H30	0.9500
I3—C3	2.079 (3)	C31—C32	1.395 (5)
F3—C6	1.356 (4)	C31—C36	1.392 (5)
C3—C4	1.383 (5)	C32—H32	0.9500
Cl4A—C44A	1.772 (12)	C32—C33	1.387 (5)
C4—C5	1.380 (5)	C33—H33	0.9500
C5—C6	1.386 (5)	C33—C34	1.389 (5)
C7—C8	1.393 (5)	C34—H34	0.9500
C7—C12	1.396 (5)	C34—C35	1.384 (5)
C8—H8	0.9500	C35—H35	0.9500
C8—C9	1.387 (5)	C35—C36	1.390 (5)
C9—H9	0.9500	C36—H36	0.9500
C9—C10	1.368 (6)	C37—C38	1.392 (5)
C10—H10	0.9500	C37—C42	1.390 (5)
C10—C11	1.366 (6)	C38—H38	0.9500
C11—H11	0.9500	C38—C39	1.396 (6)
C11—C12	1.395 (5)	C39—H39	0.9500
C12—H12	0.9500	C39—C40	1.377 (7)
C13—C14	1.397 (5)	C40—H40	0.9500
C13—C18	1.380 (5)	C40—C41	1.347 (6)
C14—H14	0.9500	C41—H41	0.9500
C14—C15	1.377 (5)	C41—C42	1.394 (5)
C15—H15	0.9500	C42—H42	0.9500
C15—C16	1.382 (6)	C43—H43a	0.9900
C16—H16	0.9500	C43—H43b	0.9900
C16—C17	1.378 (6)	C44A—H44a	0.9900
C17—H17	0.9500	C44A—H44b	0.9900
C17—C18	1.387 (5)	C44A—Cl3A	1.742 (12)
C18—H18	0.9500	Cl3B—C44B	1.72 (2)
C19—C20	1.398 (5)	C44B—H44c	0.9900
C19—C24	1.398 (5)	C44B—H44d	0.9900
C20—H20	0.9500	C44B—Cl4B	1.75 (2)
C20—C21	1.382 (5)		
			
P1—Pd1—I1	91.66 (2)	H22—C22—C21	119.8 (2)
C1—Pd1—I1	171.82 (9)	C23—C22—C21	120.4 (3)
C1—Pd1—P1	89.09 (9)	C23—C22—H22	119.8 (2)
P2—Pd1—I1	91.72 (2)	H23—C23—C22	120.2 (2)
P2—Pd1—P1	171.80 (3)	C24—C23—C22	119.7 (3)
P2—Pd1—C1	88.63 (9)	C24—C23—H23	120.2 (2)
C25—P1—Pd1	110.31 (12)	C23—C24—C19	120.4 (3)
C31—P1—Pd1	113.10 (12)	H24—C24—C19	119.8 (2)
C31—P1—C25	108.12 (16)	H24—C24—C23	119.8 (2)
C37—P1—Pd1	118.72 (11)	C26—C25—P1	123.6 (3)
C37—P1—C25	103.15 (16)	C30—C25—P1	117.2 (3)
C37—P1—C31	102.49 (16)	C30—C25—C26	119.2 (3)
C2—C1—Pd1	121.3 (2)	H26—C26—C25	120.2 (2)
C6—C1—Pd1	124.0 (2)	C27—C26—C25	119.6 (4)
C6—C1—C2	114.8 (3)	C27—C26—H26	120.2 (2)
C7—P2—Pd1	115.27 (11)	H27—C27—C26	119.6 (2)
C13—P2—Pd1	109.67 (12)	C28—C27—C26	120.9 (4)
C13—P2—C7	103.29 (15)	C28—C27—H27	119.6 (3)
C19—P2—Pd1	116.61 (12)	H28—C28—C27	120.3 (3)
C19—P2—C7	101.96 (15)	C29—C28—C27	119.5 (4)
C19—P2—C13	109.01 (15)	C29—C28—H28	120.3 (2)
C1—C2—F1	117.7 (3)	H29—C29—C28	119.8 (2)
C3—C2—F1	117.7 (3)	C30—C29—C28	120.4 (4)
C3—C2—C1	124.6 (3)	C30—C29—H29	119.8 (3)
I3—C3—C2	122.6 (2)	C29—C30—C25	120.4 (4)
C4—C3—C2	117.0 (3)	H30—C30—C25	119.8 (2)
C4—C3—I3	120.4 (2)	H30—C30—C29	119.8 (3)
C3—C4—F2	118.3 (3)	C32—C31—P1	121.1 (3)
C5—C4—F2	119.4 (3)	C36—C31—P1	119.5 (3)
C5—C4—C3	122.2 (3)	C36—C31—C32	119.5 (3)
C4—C5—I2	120.9 (2)	H32—C32—C31	119.8 (2)
C6—C5—I2	122.1 (2)	C33—C32—C31	120.4 (3)
C6—C5—C4	116.9 (3)	C33—C32—H32	119.8 (2)
F3—C6—C1	117.8 (3)	H33—C33—C32	120.1 (2)
C5—C6—C1	124.5 (3)	C34—C33—C32	119.8 (3)
C5—C6—F3	117.7 (3)	C34—C33—H33	120.1 (2)
C8—C7—P2	121.9 (3)	H34—C34—C33	120.0 (2)
C12—C7—P2	119.1 (3)	C35—C34—C33	120.1 (3)
C12—C7—C8	118.9 (3)	C35—C34—H34	120.0 (2)
H8—C8—C7	119.9 (2)	H35—C35—C34	119.8 (2)
C9—C8—C7	120.2 (3)	C36—C35—C34	120.3 (3)
C9—C8—H8	119.9 (2)	C36—C35—H35	119.8 (2)
H9—C9—C8	120.0 (2)	C35—C36—C31	119.9 (3)
C10—C9—C8	120.0 (4)	H36—C36—C31	120.1 (2)
C10—C9—H9	120.0 (2)	H36—C36—C35	120.1 (2)
H10—C10—C9	119.4 (2)	C38—C37—P1	122.6 (3)
C11—C10—C9	121.2 (4)	C42—C37—P1	118.2 (3)
C11—C10—H10	119.4 (2)	C42—C37—C38	119.2 (3)
H11—C11—C10	120.2 (2)	H38—C38—C37	120.4 (2)
C12—C11—C10	119.6 (4)	C39—C38—C37	119.3 (4)
C12—C11—H11	120.2 (2)	C39—C38—H38	120.4 (3)
C11—C12—C7	120.2 (3)	H39—C39—C38	119.7 (3)
H12—C12—C7	119.9 (2)	C40—C39—C38	120.7 (4)
H12—C12—C11	119.9 (2)	C40—C39—H39	119.7 (3)
C14—C13—P2	117.2 (3)	H40—C40—C39	120.0 (3)
C18—C13—P2	123.6 (3)	C41—C40—C39	120.0 (4)
C18—C13—C14	119.1 (3)	C41—C40—H40	120.0 (2)
H14—C14—C13	119.8 (2)	H41—C41—C40	119.5 (2)
C15—C14—C13	120.4 (3)	C42—C41—C40	121.0 (4)
C15—C14—H14	119.8 (2)	C42—C41—H41	119.5 (2)
H15—C15—C14	119.7 (2)	C41—C42—C37	119.8 (4)
C16—C15—C14	120.5 (4)	H42—C42—C37	120.1 (2)
C16—C15—H15	119.7 (2)	H42—C42—C41	120.1 (2)
H16—C16—C15	120.5 (2)	Cl2—C43—Cl1	111.3 (3)
C17—C16—C15	119.1 (4)	H43a—C43—Cl1	109.37 (16)
C17—C16—H16	120.5 (2)	H43a—C43—Cl2	109.37 (16)
H17—C17—C16	119.5 (2)	H43b—C43—Cl1	109.37 (16)
C18—C17—C16	121.0 (4)	H43b—C43—Cl2	109.37 (16)
C18—C17—H17	119.5 (2)	H43b—C43—H43a	108.0
C17—C18—C13	119.9 (3)	H44a—C44A—Cl4A	109.6 (5)
H18—C18—C13	120.0 (2)	H44b—C44A—Cl4A	109.6 (6)
H18—C18—C17	120.0 (2)	H44b—C44A—H44a	108.1
C20—C19—P2	120.9 (3)	Cl3A—C44A—Cl4A	110.2 (6)
C24—C19—P2	120.0 (3)	Cl3A—C44A—H44a	109.6 (6)
C24—C19—C20	118.9 (3)	Cl3A—C44A—H44b	109.6 (7)
H20—C20—C19	119.8 (2)	H44c—C44B—Cl3B	109.6 (6)
C21—C20—C19	120.5 (3)	H44d—C44B—Cl3B	109.6 (8)
C21—C20—H20	119.8 (2)	H44d—C44B—H44c	108.1340 (1)
H21—C21—C20	119.9 (2)	Cl4B—C44B—Cl3B	110.2 (12)
C22—C21—C20	120.1 (3)	Cl4B—C44B—H44c	109.6 (8)
C22—C21—H21	119.9 (2)	Cl4B—C44B—H44d	109.6 (8)
			
Pd1—C1—C2—F1	−0.8 (3)	C2—C3—C4—C5	0.0 (4)
Pd1—C1—C2—C3	179.9 (3)	I3—C3—C4—C5	−178.9 (3)
Pd1—C1—C6—F3	−1.0 (3)	F3—C6—C5—C4	−179.4 (3)
Pd1—C1—C6—C5	−179.9 (3)	C3—C4—C5—C6	0.1 (4)
P1—C25—C26—C27	178.6 (3)	C7—C8—C9—C10	0.8 (4)
P1—C25—C30—C29	−178.8 (3)	C7—C12—C11—C10	0.5 (4)
P1—C31—C32—C33	−178.1 (3)	C8—C9—C10—C11	−0.5 (5)
P1—C31—C36—C35	177.8 (3)	C9—C10—C11—C12	−0.1 (5)
P1—C37—C38—C39	179.7 (4)	C13—C14—C15—C16	−0.6 (5)
P1—C37—C42—C41	179.8 (3)	C13—C18—C17—C16	−0.1 (5)
F1—C2—C1—C6	178.6 (3)	C14—C15—C16—C17	−0.3 (5)
F1—C2—C3—I3	0.0 (3)	C15—C16—C17—C18	0.6 (5)
F1—C2—C3—C4	−179.0 (3)	C19—C20—C21—C22	1.8 (4)
C1—C2—C3—I3	179.2 (3)	C19—C24—C23—C22	0.3 (4)
C1—C2—C3—C4	0.3 (4)	C20—C21—C22—C23	−0.1 (4)
C1—C6—C5—I2	179.1 (3)	C21—C22—C23—C24	−1.0 (4)
C1—C6—C5—C4	−0.5 (4)	C25—C26—C27—C28	−0.1 (5)
I2—C5—C4—F2	0.8 (3)	C25—C30—C29—C28	0.4 (5)
I2—C5—C4—C3	−179.5 (3)	C26—C27—C28—C29	0.6 (5)
I2—C5—C6—F3	0.2 (3)	C27—C28—C29—C30	−0.8 (6)
P2—C7—C8—C9	−176.1 (3)	C31—C32—C33—C34	0.6 (4)
P2—C7—C12—C11	175.6 (3)	C31—C36—C35—C34	0.1 (4)
P2—C13—C14—C15	−178.1 (3)	C32—C33—C34—C35	−2.3 (4)
P2—C13—C18—C17	178.3 (4)	C33—C34—C35—C36	1.9 (4)
P2—C19—C20—C21	−177.4 (3)	C37—C38—C39—C40	1.4 (5)
P2—C19—C24—C23	176.4 (3)	C37—C42—C41—C40	−0.3 (4)
F2—C4—C3—C2	179.7 (3)	C38—C39—C40—C41	−1.7 (6)
F2—C4—C3—I3	0.7 (3)	C39—C40—C41—C42	1.2 (6)
F2—C4—C5—C6	−179.6 (3)		

## References

[bb1] Barbour, L. J. (2020). *J. Appl. Cryst.* **53**, 1141–1146.

[bb2] Bourhis, L. J., Dolomanov, O. V., Gildea, R. J., Howard, J. A. K. & Puschmann, H. (2015). *Acta Cryst.* A**71**, 59–75. 10.1107/S2053273314022207 PMC4283469 25537389

[bb3] Bruno, I. J., Cole, J. C., Edgington, P. R., Kessler, M., Macrae, C. F., McCabe, P., Pearson, J. & Taylor, R. (2002). *Acta Cryst.* B**58**, 389–397. 10.1107/s0108768102003324 12037360

[bb4] Dolomanov, O. V., Bourhis, L. J., Gildea, R. J., Howard, J. A. K. & Puschmann, H. (2009). *J. Appl. Cryst.* **42**, 339–341.

[bb5] Groom, C. R., Bruno, I. J., Lightfoot, M. P. & Ward, S. C. (2016). *Acta Cryst.* B**72**, 171–179. 10.1107/S2052520616003954 PMC4822653 27048719

[bb6] Krause, L., Herbst-Irmer, R., Sheldrick, G. M. & Stalke, D. (2015). *J. Appl. Cryst.* **48**, 3–10. 10.1107/S1600576714022985 PMC4453166 26089746

[bb7] Momose, A. A. & Bosch, E. (2010). *Cryst. Growth Des.* **10**, 4043–4049.

[bb8] Sheldrick, G. M. (2015). *Acta Cryst.* A**71**, 3–8.

[bb9] Sonogashira, K. (2002). *J. Organomet. Chem.* **653**, 46–49.

[bb10] Spackman, P. R., Turner, M. J., McKinnon, J. J., Wolff, S. K., Grimwood, D. J., Jayatilaka, D. & Spackman, M. A. (2021). *J. Appl. Cryst.* **54**, 1006–1011. 10.1107/S1600576721002910 PMC8202033 34188619

[bb11] Vicente, J., Abad, J. A., López-Serrano, J. & Jones, P. G. (2004). *Organometallics*, **23**, 4711–4722.

[bb12] Xu, J. C., Yin, Y. Z. & Han, Z. Y. (2021). *Org. Lett.* **23**, 3834–3838. 10.1021/acs.orglett.1c00910 33961444

